# Characterization of class-switched B cells in chickens

**DOI:** 10.3389/fimmu.2024.1484288

**Published:** 2024-11-21

**Authors:** Dominik von La Roche, Magdalena Schumacher, Marina Kohn, Johanna Trapp, Benjamin Schusser, Silke Rautenschlein, Sonja Härtle

**Affiliations:** ^1^ Department of Veterinary Sciences, AG Immunology, Ludwig-Maximilians-Universität München, Planegg, Germany; ^2^ Clinic for Poultry, University of Veterinary Medicine Hannover, Hannover, Germany; ^3^ Reproductive Biotechnology, TUM School of Life Sciences, Technische Universität München, Freising, Germany; ^4^ Center of Infection Prevention (ZIP), Technische Universität München, Freising, Germany

**Keywords:** chicken, B cell differentiation, Ig class switch, CD40L, IL-10, vaccination

## Abstract

While B cell development in the birds’ primary B cell organ, the bursa Fabricius, is relatively well understood, very little is known about post bursal B cell differentiation into plasma and memory cells though these cells are essential for a protecting antibody response and so far, no specific markers for these cells were available. Since immunoglobulin class switch is one part of the B cell differentiation process, our objective was to conduct a first detailed investigation of class-switched chicken B cells. As only very few IgY and IgA expressing cells were detected in lymphoid organs of young chickens, we used CD40L and IL-10 to establish a prolonged *in vitro* culture system, which induces B cell proliferation, class switch to IgY and IgA and enhanced antibody secretion. This enabled a phenotypic analysis of differentiating B cells. Importantly, these cells lost surface expression of the B cell markers chB6 and BAFF-R. B cell receptor surface expression remained unchanged, showing that while differentiating toward plasma cells, B cells can be addressed by L chain staining. Newly generated potential plasma cell markers CD138 and TACI showed only a transient expression on cultured cells and rather act as markers for B cell activation than plasma/memory cells in general. CD57 upregulation was connected to activation and blast formation but not to class switch. We also examined potential changes in class-switched cells in different age groups and post vaccination. Surprisingly, bursa involution, laying and age had no distinct effects on the presence of class-switched cells, but we detected significantly more class-switched B cells post vaccination. Hence, we are now able to generate class-switched plasmablasts *in vitro* for a more detailed characterization and can address them under different conditions in chickens for further analysis of their B cell response.

## 1 Introduction

Vaccination is an essential measure to protect commercial poultry flocks against a multitude of pathogens. The mode of action of many vaccines is the induction of a protective antibody response (1, 2). Hence, to improve vaccine design and application, a better knowledge on antibody secreting cells (ASCs) and B cell memory in the chicken is indispensable. As the chickens B cell system differs substantially from that in human and mice, knowledge from these species can be transferred only to a very limited extent. The most striking difference is the bursa of Fabricius, a gut associated lymphoid tissue, which serves as the avian primary B cell organ (3). In addition, chickens have no lymph nodes, which strongly limits the number of secondary lymphoid tissues and the potential locations for B cell – T cell-contact. In the spleen, no separation between marginal and follicular B cells is visible and the differently structured germinal centers (GC) are located in the T cell area (4). Furthermore, the bursa involutes with sexual maturity, ceasing the production of new B cells in the primary B cell organ (5). Consequently, the maintenance of the peripheral B cell pool is left to a largely unknown mechanism (6, 7).

Whereas the processes of B cell receptor (BCR) repertoire generation in the bursa are relatively well understood (8), there is still only scarce information on the differentiation processes happening in the periphery after the cells have left the bursa. Chicken plasma cells, as finally differentiated ASCs, have been identified by immunohistochemistry on the basis of their typical morphology as large cells with an eccentric nucleus with cartwheel structure and a strong intracellular staining for immunoglobulins (8). But only very few reports exist about their differentiation from naïve B cells (9) and their isolation (10) for a more detailed characterization. For chicken B memory cells, the only prove of their existence so far is the successful induction of enhanced antibody recall reactions by prime - boost vaccination (11) and immunoglobulin class switching.

The phenotype of mammalian memory and plasma cells can be addressed by various markers like CD27 for memory cells (12) and CD138 for plasma cells (13, 14). In addition, during the differentiation process a switch in the expression of the three receptors for B cell activating factor of the TNF family (BAFF) occurs: while immature and mature B cells express BAFF-R, activation and differentiation induces a switch to the expression of transmembrane activator and calcium-modulator and cytophilin ligand interactor (TACI) (15–17) and plasma cells additionally express the B cell maturation antigen (BCMA) (18, 19).

Before chicken B cell precursors migrate to the bursa, they start to express the chB6 antigen, a highly glycosylated type I transmembrane protein (20). Subsequently, both bursal and peripheral chicken B cells can be addressed by their expression of chB6 (21, 22). However, plasma cells have been shown by immunohistochemistry to lose the chB6 antigen to an undetectable level (21), an observation which has been confirmed by *in vitro* induced plasmablast like cells, which had a strongly reduced chB6 expression (23). Bursal and peripheral chicken B cells express BAFF-R (24–26), which can be addressed by a specific antibody (27). A chicken homologue of TACI was also identified (24), but surface expression of TACI protein was not yet analyzed. Though a chicken BCMA gene was identified, it was shown to be a pseudogene (24, 28). Hence, if a differentiation-stage specific switch in the expression of BAFF receptors occurs on chicken B cells, it must follow a different pattern than that in mammals, due to the expression of only two receptors.

Initially, all naïve B cells express an IgM type BCR. One of the events, which can occur during differentiation of a B cell in the GC is immunoglobulin (Ig) class switch from IgM to other Ig isotypes (29). Class-switched B cells can therefore be regarded as differentiated cells. In chickens the repertoire Ig isotypes is limited (30–32) and class switch can only occur from IgM toward IgY, described as intermediate between IgG and IgE (31, 33) or to IgA, the dominant Ig of mucosal surfaces (34–36).

In contrast to human blood, where age dependent up to 25% of blood B cells have undergone class switch (37–39), the vast majority of peripheral chicken B cells was reported to express IgM (40, 41), making other isotypes infrequent. For a better access to these rare cells, an *in vitro* culture system could be helpful.

B cell activation includes two main signals: antigen binding to the BCR and the binding of CD40 by CD40L, which is provided by activated T helper cells (42). Fine adjustment of the reaction is obtained by the response to additional cytokines like IL-4, IL-6, IL-10 and IL-21 (43–48) which direct B cell fate toward memory or plasma cells and the type of class switch. *In vitro*, strong CD40- signaling induced by recombinant CD40L can be sufficient for B cell activation (23, 49). In cultures of human B cells, addition of IL-10 to CD40L activated B cells leads, depending on the duration and sequence of stimulation, to the differentiation into a memory or plasma cell phenotype (46) and induces class switch to IgG (50).

Using cultures of chicken B cells, we have shown that recombinant CD40L drives them toward a plasma cell phenotype and enhances antibody production (23). The availability of recombinant chicken IL-10 (51) prompted us to test its potential for B cell differentiation *in vitro*.

We established and characterized a culture system which enables Ig class switch and antibody secretion. Based on the gained knowledge we have examined the presence of class-switched B cells pre- and post-bursal involution and post IBDV vaccination.

## 2 Materials and methods

### 2.1 Animals

Fertilized eggs of M11 white layer chicken (B2/B2 haplotyp) were provided by Prof. Dr. Steffen Weigend (FLI Mariensee) and incubated and hatched at the Faculty of Veterinary Medicine, LMU Munich. Animals did not receive vaccinations except if required for experiments. The animals were housed in groups under conventional conditions and received both food and water ad-libitum. Chickens of the layer-line Lohmann-Sandy were kept under conventional conditions on different organic farms and vaccinated following standard vaccination protocols for poultry. Experiments with M11 birds were performed at the age of 16-24 weeks, animals of the Lohmann-Sandy chicken were 7, 17, 27 and 70 weeks old.

For the IBDV vaccination experiment 6-week-old M11 chickens were vaccinated orally with one dose of Nobilis ^®^ Gumboro 228E (Intervet Germany GmbH, Unterschleißheim, Germany), as defined by the manufacturer.

### 2.2 Cell preparation

The animals were euthanized through exsanguination following anesthesia. Organs were aseptically dissected and placed in transport media (RPMI 1640, Thermo Fisher Scientific, Waltham, USA) supplemented with 1% Penicillin-Streptomycin (Merck KGaA, Darmstadt, Germany) at room temperature (RT) until further processing.

Heparinized blood was collected from the jugular vein, bone marrow (BM) was acquired by flushing it from the femoral bone. Single cell suspensions of BM, spleen and caecal tonsil were obtained by mechanical dissociation of the tissue through a stainless-steel. Subsequently, leukocytes were separated using density gradient centrifugation with Histopaque^®^-1077 (Merck KGaA, Darmstadt, Germany). All steps involving tissue preparation and centrifugation were conducted at room temperature to maintain optimal cellular viability and integrity for chicken B-cells.

### 2.3 Cell culture and stimulation

Splenocytes were cultured in 24-well-plates with 2.5x10^6^ c/ml in IMDM (Thermo Fisher Scientific, Waltham, USA) supplemented with 8% FBS Superior (Merck KGaA, Darmstadt, Germany), 2% chSerum ((Thermo Fisher Scientific, Waltham, USA) and 1% Penicillin-Streptomycin (Merck KGaA, Darmstadt, Germany) at 40°C with 5% CO_2_. Cell culture supernatant produced by a bioreactor containing soluble recombinant cytokines (CD40L and IL-10) were added at a concentration of 2.5%. To maintain optimal cell growth and viability half of the medium was replaced every second day and cells were split into two wells at the third day of culture.

### 2.4 Recombinant cytokines

HEK293 cells were stably transfected with a trimerized chCD40L-muCD8 construct that has been previously described (23, 52). Clones exhibiting the highest production rates were selected for further use and cultured in a WHEATON^®^ CELLine™ bioreactor (DKW Life Sciences, Wertheim, Germany) for adherent cells with RPMI (Thermo Fisher Scientific, Waltham, USA), supplemented with 20% FBS (Merck KGaA, Darmstadt, Germany). Bioreactor supernatant was sterile filtered and titrated to determine the optimal concentration.

For rchIL10, a pCI-neo-chIL-10 expression construct was generously supplied by Dr. Pete Kaiser (Roslin Institute, Edinburgh, UK) (51). Subsequently, the chIL10 sequence was cloned into a modified pCR3 vector containing a flag-sequence (kindly provided by Dr. Pascal Schneider, Lausanne, Switzerland) by using primers (**Table 1**) to introduce PstI and EcoRI restriction sites. The resultant chIL10-Flag construct was then stably transfected in HEK293 cells, and the clone demonstrating the highest production rate was subsequently cultured under selective conditions in a WHEATON^®^ CELLine™ 1000AD bioreactor (DKW Life Sciences, Wertheim, Germany) with RPMI (Thermo Fisher Scientific, Waltham, Massachusetts, USA) supplemented with 10% FBS (Merck KGaA, Darmstadt, Germany) and 250µg/ml Genticin (G418) (Carl Roth GmbH + Co.KG, Karlsruhe, Germany). To obtain purified rChIL-10, cell culture supernatants were purified by affinity chromatography using an anti-Flag-M2 column (Merck KGaA, Darmstadt, Germany). After initial experiments with purified protein, cell culture supernatants were titrated against the purified protein to determine the optimal concentration and used for further experiments.

**Table 1:** Forward and reverse primers used for qRT-PCR.

Gen	Forward Primer	Reverse Primer
***chIL10***	^5’^ATATCTGCAGTGCACCCTGCCTGCCCA^3^’	^5’^GCGCGAATTCTCACTTCCTCCTCCTC^3’^
***chCD138***	^5’^TAGAATTCAACCTTCCTCCTGAAGATCTC^3^	^5^’TACTCGAGTTATGCATAGAACTCTTCTTG^3’^
***chTACI***	5’ATGGACGGGACGTGCCTGGGAAGAGC3’	5’GCACCATGGGCGTCTTCTCTTGC3’
***RPL13***	^5’^GAGGTGCCCGACTGTCAGAT^3’^	^5’^ATCGTCCGAGCAAACCTTTTGT^3’^
***chB6***	^5’^GATCGCCTGCCCTCCAAT^3’^	^5’^TGGCTTTCCACGTCAGCTATC^3’^
***CD79b***	^5’^ GCGTCCCCATGCTCCTCTTCCTA^3’^	^5’^GCAGCACCCCTCACTCCTCTCCT^3’^
***PAX5***	^5’^CCAGCAGCAGTTGGAAGTGTT^3’^	^5’^CTCTGGTTTGATGGGCTCTGTT^3’^
***µS***	^5’^GGAGAACCCCGAAAATGAGT^3’^	^5’^GCCAACACCAAGGAGACATT^3’^
***BLIMP1***	^5’^GTGGTATTGCCGAGACTTTGC^3’^	^5’^GGGTTTGTGTGAGGTTCATCATT^3’^
***BCL6***	^5‘^GAAGACCCCAAGGGAAGAGTTT^3’^	^5‘^CTGAGACATCTCTGCCTCGATAAG^3‘^
***AICDA***	^5‘^CGTCTGAAACCCAGCAAGAGT^3’^	^5’^TGTCCATGTCAGCTGGGTTCT^3’^

### 2.5 [^3^H]-Thymidine assay

To detect cell proliferation, 1x10^6^ isolated spleen leukocytes were cultured in 96-well-plates in the presence and absence of CD40L, IL-10 and the combination of both cytokines at 40°C with 5% CO_2_. Medium served as control. After 48 hours cells were pulsed with [3H]-Thymidine (GE HealthCare, Amersham, UK) and harvested 16 hours later.

### 2.6 mAbs for chCD138 and chTACI

For the generation of an anti-CD138 (Syndecan 1) antibody, we amplified the chCD138 full length sequence (ENSGALT00010019066.1) from Harderian gland cDNA (see **Table 1** for primers). To generate an anti-chTACI mAb, we amplified the full length sequence from pcTACI, a construct of chTACI in pcIneo, kindly provided by Dr. John Young (24) (Institute for Animal Health, Compton, UK) (see **Table 1** for primers). The PCR products were ligated into pcDNA 3.1/CT-GFP-TOPO (Thermo Fisher Scientific, Waltham, USA). Successful cloning was confirmed by restriction enzyme digest and subsequent Sanger sequencing. HEK293 cells were stably transfected and selected for green fluorescent protein (GFP) fluorescence. BALB/c mice were immunized three times with 1x10^8^ HEK293-chCD138-GFP or HEK293-chTACI-GFP.

Murine spleen cells were fused to SP2/0-Ag14 cells, and supernatants of resulting hybridomas were tested by flow cytometry on transfected and untransfected HEK293 cells. The selected hybridoma 7G10 for chCD138 is an IgG1, the selected hybridoma 1H4 for chTACI an IgG2b antibody.

### 2.7 Flow cytometry, cell sorting and cell counting

Staining of cells for flow cytometric analysis was performed according to standard procedures. Briefly, 10^6^ cells were transferred to a 96-well plate and incubated with Fixable Viability Dye eFluor™ 780 dilution (Thermo Fisher Scientific, Waltham, USA). Subsequently, cells were stained with 50µl of monoclonal antibody (mAb) mixture (see **Table 2**). mAbs were either directly conjugated or detected by fluorochrome conjugated secondary antibodies (**Table 3**). If necessary, when the unconjugated primary antibodies and the direct conjugates were of the same isotype, a blocking step with normal mouse serum (Jackson ImmunoResearch, West Grove, USA) was interposed before applying the directly conjugated antibody.

**Table 2:** Primary mAbs and directly conjugated mAbs.

Antigen	Clone	Species	Isotype	Fluorchrome	Source of antibody
chB6	AV20	Mouse	IgG1		Southern Biotech
chB6	AV20	Mouse	IgG1	Pacific blue	Southern Biotech
chB6	AV20	Mouse	IgG1	RPE	Southern Biotech
chBAFF-receptor	2C4	Mouse	IgG1		(27)
chCD138	7G10	Mouse	IgG1		this manuscript
chCD25	28-4	Mouse	IgG3		(118)
chCD28	2-4	Mouse	IgG2a		(119)
chCD4	CT4	Mouse	IgG1	FITC	Southern Biotech
chCD8	3-298	Mouse	IgG2b		(120)
chCD80	AV82	Mouse	IgG2a		The Pirbright Institute
chCXCR4	9D9	Mouse	IgG2a		(121)
chCXCR5	6A9	Mouse	IgG1		(122)
chMHCII	2G11	Mouse	IgG1		Southern Biotech
chTACI	1H4	Mouse	IgG2b		this manuscript
Human CD57	NK-1	Mouse	IgM	APC	BD-Bioscience
chL chain	2G1	Mouse	IgG1	CF405M	Bio-Rad
IgA	A1	Mouse	IgG2b		Southern Biotech
IgA	K3	Mouse	IgG2a		(123)
IgA	K3	Mouse	IgG2a	Biotin	(123)
IgM	M1	Mouse	IgG2b		Southern Biotech
IgM	M1	Mouse	IgG2b	Biotin	Southern Biotech
IgY	G1	Mouse	IgG1		Southern Biotech
IgY	4D12	Mouse	IgG1	Biotin	(123)
TCRγδ	TCR1	Mouse	IgG1		Southern Biotech

**Table 3:** Fluorochrome conjugated secondary antibodies.

Antigen	Fluorchrome	Source of antibody
Mouse-IgG1	APC	Jackson Laboratories
Mouse-IgG1	FITC	Southern Biotech
Mouse-IgG1	BV421	Jackson Laboratories
Mouse-IgG2a	FITC	Southern Biotech
Mouse-IgG2a	Alexa Fluor 647	Thermo Fisher scientific
Mouse-IgG2b	RPE	Southern Biotech
Mouse-IgG3	FITC	Southern Biotech
Mouse-IgG3	Alexa Fluor 647	Southern Biotech
Streptavidin	APC	Jackson Laboratories

All incubation steps were carried out for 20 minutes in the dark on ice. In between the incubation steps, cells were washed with 200µl of staining buffer (PBS pH 7.2 (Thermo Fisher Scientific, Waltham, USA), 1% bovine serum albumin and 0.1% NaN_3_ (ITW Reagents, Castellar del Vallès, Spain)).

The anti-chicken L chain mAb (**Table 2**) was conjugated to CF405M using the Mix-n-Stain™ CF™ 405M Antibody Labeling Kit (Merck KGaA, Darmstadt, Germany).

For optimal staining results, all used mAbs and fluorochrome-conjugated secondary antibodies were titrated to their optimal dilution prior to the experiments.

For analysis of different tissues, 100.000 viable CD45^+^ single cells were recorded per sample, for analysis of *in vitro* cultures, 50.000 viable single cells.

Flow cytometry was performed with a FACSCanto (Becton Dickinson, Heidelberg, Germany) and data were analyzed using FACSDiva (Becton Dickinson, Heidelberg, Germany) and FlowJo (FlowJo LLC, Oregon, USA) software, version 10.10.0.

Sort purification of cells was performed on a FACSAria Illu (Becton Dickinson, Heidelberg, Germany) with FACS DIVA software.

The number of living cells was determined by trypan blue exclusion, and the frequency of living cells, negative for Fixable Viability Dye eFluor™ 780 (Thermo Fisher Scientific, Waltham, USA), was analyzed by flow cytometry as described above. Absolute numbers of viable cells were calculated from these values.

### 2.8 qRT-PCR

For qRT-PCR, RNA of sort purified L chain positive/CD80 positive and L chain positive/CD80 negative splenic leukocytes, was prepared using the ReliaPrep™ RNA Miniprep Systems kit (Promega, Madison, USA) following the manufacturer’s instructions.

The RNA was quantified on a NanoDrop ND-1000 (Thermo Fisher Scientific, Waltham, MA, USA), and RNA quality was determined on a 2100 Bioanalyzer (RIN > 9) (Agilent Technologies, Santa Clara, CA, USA). cDNA was prepared using the GoScript™ Reverse Transcriptase kit (Promega, Madison, USA) according to manufacturer’s instruction and analyzed for the relative abundance of *RPL13*, *chB6*, *CD79b*, *µS*, *BCL6*, *PAX5*, *BLIMP1* and *AICDA* using primers from Eurofins Genomics Germany GmbH (Ebersberg, Germany) specified in **Table 1** and the GoTaq^®^ qPCR MasterMix (Promega, Madison, USA).

Quantitative RT-PCR was performed using a 7300 Real-Time PCR System^®^ (Applied Biosystems, Warrington, UK) with SYBR-green.

Relative expression fold change for the respective genes was assesses using normalized cycle threshold values (ΔCT). Normalization was carried out against the expression in sort purified L chain^+^/CD80^-^ cells of unstimulated splenic leukocytes.

### 2.9 ELISpot assays

On the day preceding the experimental procedure, nitrocellulose ELISpot microtiter plates (Merck KGaA, Darmstadt, Germany) were coated with 100µl of anti-chL chain antibody solution (5µg/ml in sterile PBS, pH 7.2 (Thermo Fisher Scientific, Waltham, USA)), sealed with an adhesive foil, and subsequently incubated overnight at 4°C. Following this incubation, any unbound antibody was removed by washing the plates with PBS. Subsequently, the wells were treated with a blocking solution consisting of PBS supplemented with 10% FBS and incubated for two hours at 40°C.

Blocking-buffer was discarded and the residual liquid was tapped out onto cellulose.

Cytokine-stimulated splenic leukocytes were isolated via density gradient centrifugation and adjusted to a density of 1x10^5^ for IgM and 2x10^5^ cells/ml for IgA and IgY analysis. Subsequently, 100µl of this cell suspension was applied to each well of the plate. The plate was then incubated at 40°C with 5% CO_2_ for a duration of 48 hours.

Following the incubation period, a triple washing step with PBS containing 0.01% Tween^®^-20 (ITW Reagents, Castellar del Vallès, Spain) was conducted to remove any nonspecifically bound material.

Biotin (Roche, Basel, Switzerland) was used to biotinylate Ig-specific antibodies (**Table 2**) and the antibodies were then added to the plate and allowed to incubate for one hour at room temperature in the dark. After another triple washing step with PBS-T, peroxidase-conjugated streptavidin was applied to the plate as the detection antibody.

After a further washing step with PBS, 50µl of KPL TrueBlue™ Substrate (LGC Clinical Diagnostics, Teddington, UK) was added to each well and allowed to incubate for five minutes. The reaction was terminated upon the appearance of intense blue staining in the spots by discarding the remaining substrate and promptly washing the plate with distilled water.

Antibody secreting cells, visible as blue spots, were analyzed using the Bioreader^®^ 7000 V machine (BIOSYS Scientific Devices GmbH, Karben, Germany) with the EazyReader software (Version 20.9, Patch 143) that allows for automated counting of the spot number as well as the analysis of total optical density (TOD = integral accumulation of area and intensity of each spot).

### 2.10 IBDV ELISA

The presence of IgY antibodies specific to the IBDV VP2 protein was assessed in serum samples using a commercial indirect ELISA kit (ID Screen^®^ IBD VP2, Innovative Diagnostics, France). Antibody titers were calculated according to the manufacturer’s instructions. The IBD immune status is considered positive if the antibody titer is greater than or equal to the cut-off value of 1002.

### 2.11 Statistics

Data were analyzed using one-way or two-way ANOVA with subsequent Bonferroni multiple comparisons in GraphPad Prism 5.04 (GraphPad Software, Boston, USA).

## 3 Results

### 3.1 sIg class-switched cells are hardly detectable in non-mucosal tissues

**Figure 1:**
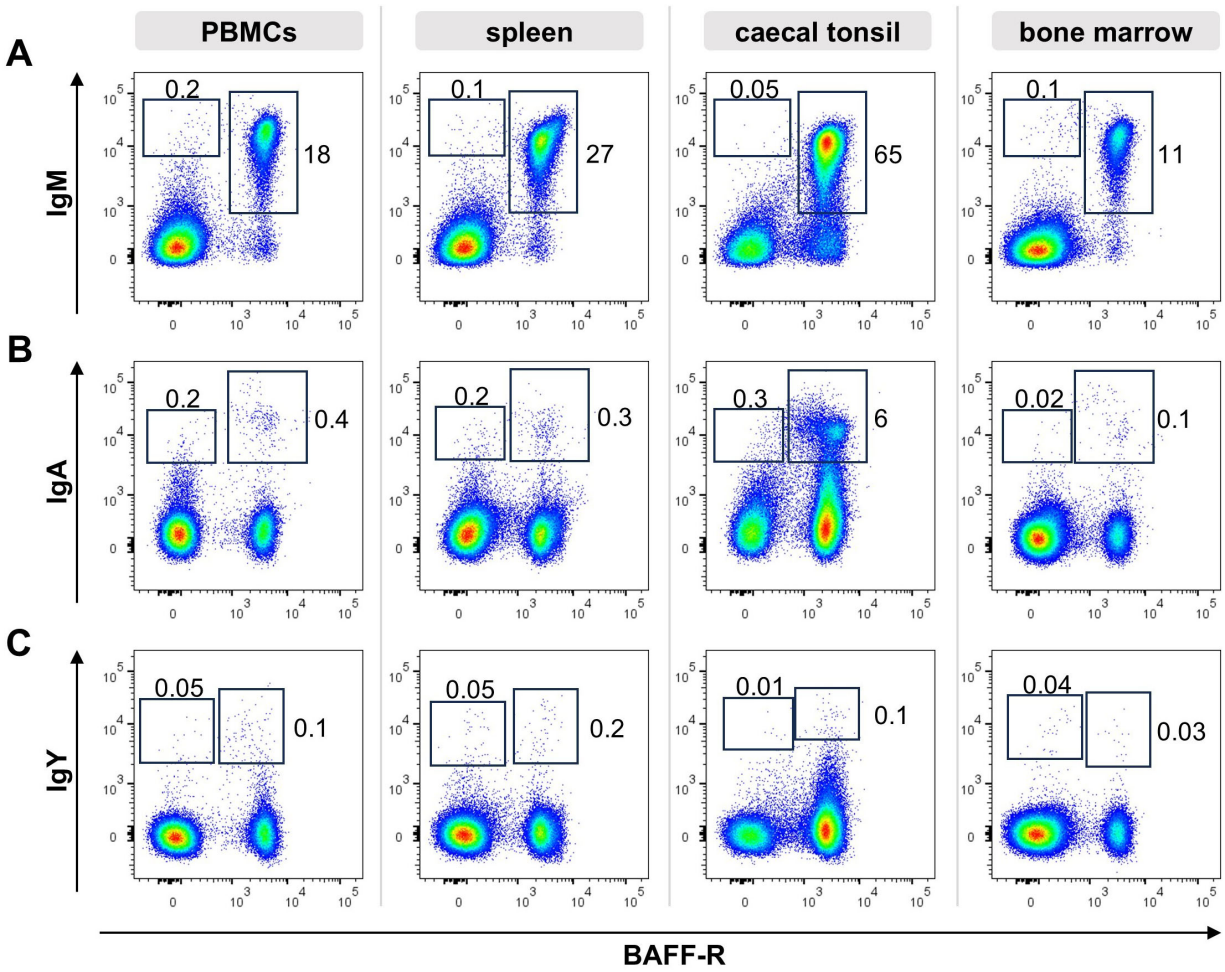
Ig surface expression in lymphoid tissues. Leukocytes were isolated from the respective tissues of a young adult chicken and analyzed by flow cytometry for the expression of surface IgM **(A)**, IgA **(B)**, IgY **(C)** and BAFF-R. Blots are gated for viable, single CD45^+^ lymphocytes. Data are representative for four independent experiments.

So far, no specific markers for chicken plasma and memory B cells were described. Since class switch of the B cell receptor (BCR) can be one part of the B cell differentiation process and antibodies for all three chicken immunoglobulin (Ig) isotypes are available, we started our search for differentiated peripheral B cells by flow cytometric analysis of Ig expression on B cells from blood, spleen, caecal tonsil and bone marrow. Therefore, cells were stained for CD45 as a pan-leukocyte marker, the B cell marker BAFF-R and IgM, IgA or IgY and at least 100,000 CD45^+^ viable single cells were counted (**Figure 1**).

In all tissues, the vast majority of B cells expressed surface IgM (sIgM), (96% of B cells in the blood, 98% in the spleen, 87% in the caecal tonsil and 98% in the bone marrow, **Figure 1A**). However, we consistently detected a small but distinct population of BAFF-R^+^ B cells expressing surface IgA (sIgA) with 3%, 1,4% and 1,6% in blood, spleen and bone marrow. With 12,7%, B cells from the gut associated caecal tonsil contained a much higher amount of IgA expressing B cells (**Figure 1B**). Although, IgY is the dominant immunoglobulin in chicken serum (53–55), no distinct IgY^+^ B cell population was detectable in the analyzed tissues. In spleen and bone marrow a few IgY^high^ cells were found, but other positive cells presented largely as a IgY^low^ smear above the isotype control background (**Figure 1C**). Mean frequencies of sIg^+^/marker ^+/-^ cells are shown in **Supplementary Table 1**.

Most of the surface Ig positive cells were also positive for the BAFF-receptor, defining them as B cells. However, to a variable extent in all tissues and for all three immunoglobulins, additional Ig^low^/BAFF-R^-^ cells were detected. In order to verify this observation, the staining was repeated with a second B cell marker chB6, which also demonstrated chB6^-^ BCR^+^ cells (**Supplementary Figure 2**). Hence, based on the expression of the B cell markers BAFF-R and chB6, two different Ig positive populations can be discriminated: i) (B cell) marker-positive, Ig positive cells and ii) marker-negative/Ig positive cells. As immunohistochemistry had shown chicken plasma cells as Bu1 negative cells or as cells that show only very weak staining (21), the later population could contain plasma cells.

It should be noted that BAFF-R and chB6 staining in the spleen and caecal tonsil were not entirely homogeneous. In many animals, a small, variably well-defined subpopulation with higher expression levels was observed (**Supplementary Figure 2**).

### 3.2 Establishment of a CD40L/IL-10 based *in vitro* culture system for B cell differentiation

As the frequency of class-switched cells among freshly isolated B cells was quite low and the origin of BAFF-R^-^/Bu1^-^/Ig^+^ cells remained unclear, we aimed to differentiate chicken B cells *in vitro* to follow the differentiation process and get enough cells for phenotyping. Therefore, we combined CD40L, which was already shown to induce *in vitro* proliferation of chicken B cells (23) with chicken IL-10.

#### 3.2.1 CD40L and IL-10 act synergistically on short term proliferation

**Figure 2:**
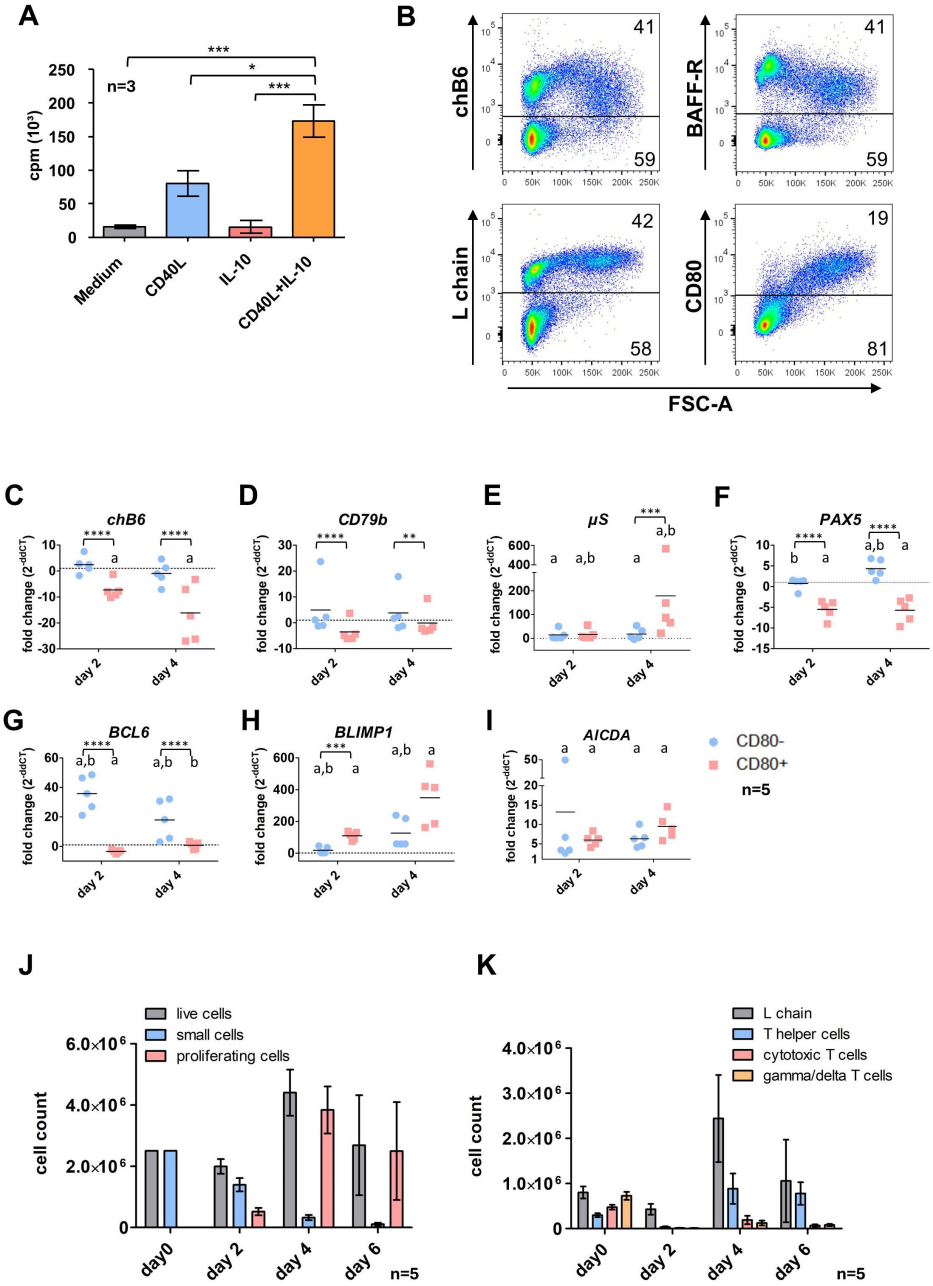
Effects of CD40L and IL-10 stimulation on spleen leukocytes. **(A)** Splenic leukocytes were isolated and incubated in the presence of the respective cytokines or medium only. After 48h [³H]-Thymidine was added and cultures were harvested 16h later to assess proliferation. Shown are mean ± SD, with*: P ≤ 0.05; ***: P ≤ 0.001 by two-way ANOVA with Bonferroni *post hoc* test. Shown are mean ± SD of three independent experiments. **(B)** Cells were stimulated for 48h with CD40L/IL-10 and analyzed by flow cytometry. Blots are gated for viable, single leukocytes. **(C–I)** L chain^+^/CD80^+^ and L chain^+^/CD80^-^ cells were sort purified from CD40L/IL-10 stimulated cultures after 2 and 4 days. Expression of the respective genes was determined by qRT-PCR and normalized to freshly isolated, sort-purified L chain^+/^CD80^-^ cells by the 2^ΔΔCT^ method. Shown are individual values and mean of five animals. Statistics were calculated by two-way ANOVA with Bonferroni *post hoc* test on ΔCT values. *Asterisks indicate statistical differences between the groups at a single time point, while letters indicate statistical differences within the population between the different time points.* *: p ≤ 0.05; **: p ≤ 0.01; ***: p ≤ 0.001; ****: p ≤ 0.0001; a: p ≤ 0.05 compared to d0; b: p ≤ 0.05 comparison between day2/day4. **(J, K)** Splenic leukocytes were stimulated with CD40L/IL-10 for 6 days. At the indicated timepoints, cells were analyzed by flow cytometry populations according to size (J, gated for viable, single leukocytes) or surface marker expression (K, gated from viable, single leukocytes (day 0) and viable, single proliferating leukocytes (d2-6)). Helper T cells were addressed as CD4^+^ cells, cytotoxic T cells as CD8^+^/TCR1^-^ and γδ T cells as TCR1^+^. Shown are mean ± SD of five animals.

Analysis of [³H]-thymidine uptake in splenocyte cultures 48h post stimulation showed that IL-10 alone did not induce any cell proliferation. Stimulation with CD40L led to substantial proliferation but the combination of both CD40L and IL-10 resulted in a significant synergistic effect (**Figure 2A**). Titration for optimal concentration of IL-10 is depicted in **Supplementary Figure 3**.

The synergistic effect of both cytokines was also seen when cultures were analyzed by flow cytometry. Here, after 48h the fraction of blasting FSC^high^ (proliferating) cells in CD40L and IL-10 stimulated cultures was almost 3fold higher than with CD40L alone (33% vs. 13%). In addition, staining for L chain demonstrated that after CD40L/IL-10 stimulation more than 90% of FSC^high^ cells are L chain^high^ B cells (**Figure 2B**, the corresponding CD3 staining is shown in **Supplementary Figure 6**).

#### 3.2.2 Early transcriptomic and phenotypic changes by CD40L and IL-10 stimulation

Flow cytometric analysis of CD40L/IL-10 stimulated splenocytes after 48h showed a decreased expression of the two B cell markers chB6 and BAFF-R within proliferating FSC^high^ cells compared to the non-proliferating subset. We also found cells of medium FSC with high chB6 and low BAFF-R expression, which were most likely derived from the small chB6^high^ subpopulation, which was present in these birds.

Strikingly, as demonstrated by the largely identical L chain staining between small and large cells, expression of the BCR was not affected and L chain positive cells represented the vast majority of proliferating blasts. In addition, CD80 was strongly upregulated on proliferating cells and this costimulatory molecule was exclusively found on FSC^high^ cells (**Figure 2B**).

Based on this correlation of size increase and CD80 expression, we sort purified chB6^+^/CD80 positive (large) and chB6^+^/CD80 negative (small) B cells after 2 days and also after a longer culture of 4 days (**Figures 2C–I**) and analyzed the expression of selected markers for B cell differentiation and stage specific transcription factors by qRT-PCR in comparison to freshly isolated splenic B cells.

In general, strong differences were detected between small and blasting cells and also for both populations in comparison to fresh cells over time.

As shown for the protein by flow cytometry, *chB6* mRNA abundance was likewise reduced in activated CD80^+^ cells compared to CD80^-^ at day 2 and showed further strong reduction in CD80^+^ blasts at day 4 (**Figure 2B**).

No significant effects of CD40L/IL10 stimulation were detected of the BCR component *CD79b*, when groups were compared over time. However, as the tendency to higher (CD80^-^) and lower (CD80^+^) expression was oppositional, differences on the ΔCT level between small and blasting cells were significant. This picture does not exactly meet the increased L chain surface staining of proliferating cells, but confirms that stimulated cells do not loose BCR surface expression (**Figure 2B**).

*µS*, the secretory form of IgM was increased in both groups at day 2 and in day 4 CD80^-^ cells (7, 10 and 6fold) and showed a strong 100fold increase in CD80^+^ cells at day 4 (**Figure 2E**).

Among the analyzed transcription factors, the expression of both the B cell-specific transcription factor *PAX5* and the germinal center/memory cell-associated transcription factor *BCL-6* were reduced in CD80^+^ cells (**Figures 2F, G**), while they remained fairly constant (*Pax5*) or showed a strong upregulation (*BCL-6*) in the CD80^-^ population. In contrast, *BLIMP1*, the plasma cell specific transcription factor, revealed the opposite profile and was strongly upregulated in CD80^+^ cells (**Figure 2H**).

Regarding its essential role for class switch recombination, we also measured expression of *AICDA*. We found a significantly increased *AICDA* abundance in all groups compared to day 0, but between small and blasting cells no significant differences were detected (**Figure 2I**).

#### 3.2.3 Cell composition in extended cultures

Prompted by these results and the knowledge that in cultures stimulated solely with CD40L the plasmablast phenotype becomes more prominent after a longer period of cultivation (23), we extended the culture of CD40L/IL-10 stimulated cells up to six days.

To examine the cellular composition of these total spleen leukocyte cultures, cells were analyzed by flow cytometry every second day. While in the first two days the frequency of living cells dropped to around 45% (data not shown), the number of viable cells was only reduced to 75% of the starting material and about one quarter of the cells presented as proliferating blasts. While small cells were continuously lost from culture, the number of blasting was strongly increased at day 4 and day 6 (representing 90% of all cells), and consequently also the total number of living cells (**Figure 2J**).

As we started with a mixed culture of splenic B and T cells, we examined the cultures for the presence of different lymphocyte populations among the blasting cells. As shown in **Figure 2K** after 2 days, the blast population contained around 90% B cells and only few proliferating helper, cytotoxic and γδ T cells were found. From day 2 to day 4 of culture B cell blasts increased 6fold in number (from 4x10^5^ to 2,4 x10^6^) indicating strong proliferation among these cells.

Strikingly, T helper cell numbers show a 25fold increase between day 2 and day 4 and hence, these cells exhibit the highest proliferation rate at this timepoint and at day 6, cultures contained roughly the same amount of L chain and CD4^+^ cells. In addition, we also found that cytotoxic and yd T cells start to proliferate and between day 2 and day 4 their numbers increased 14 and 10-fold, respectively.

Except for certain T helper cell cultures, most cell populations exhibited a decrease in cell numbers by day 6. In the case of B cell blasts, this decline was observed across all cultures, although the extent varied significantly, ranging from 70% to 16% of the cell count on day 4. This reduction may be partially attributed to suboptimal growth conditions following the proliferation peak on day 4.

Hence, extended culture periods after CD40L/IL-10 stimulation led to an initially strong growth of B and to later timepoints also induced T cell proliferation.

#### 3.2.4 CD40L/IL-10 induces both increased Ig production and class switching

**Figure 3:**
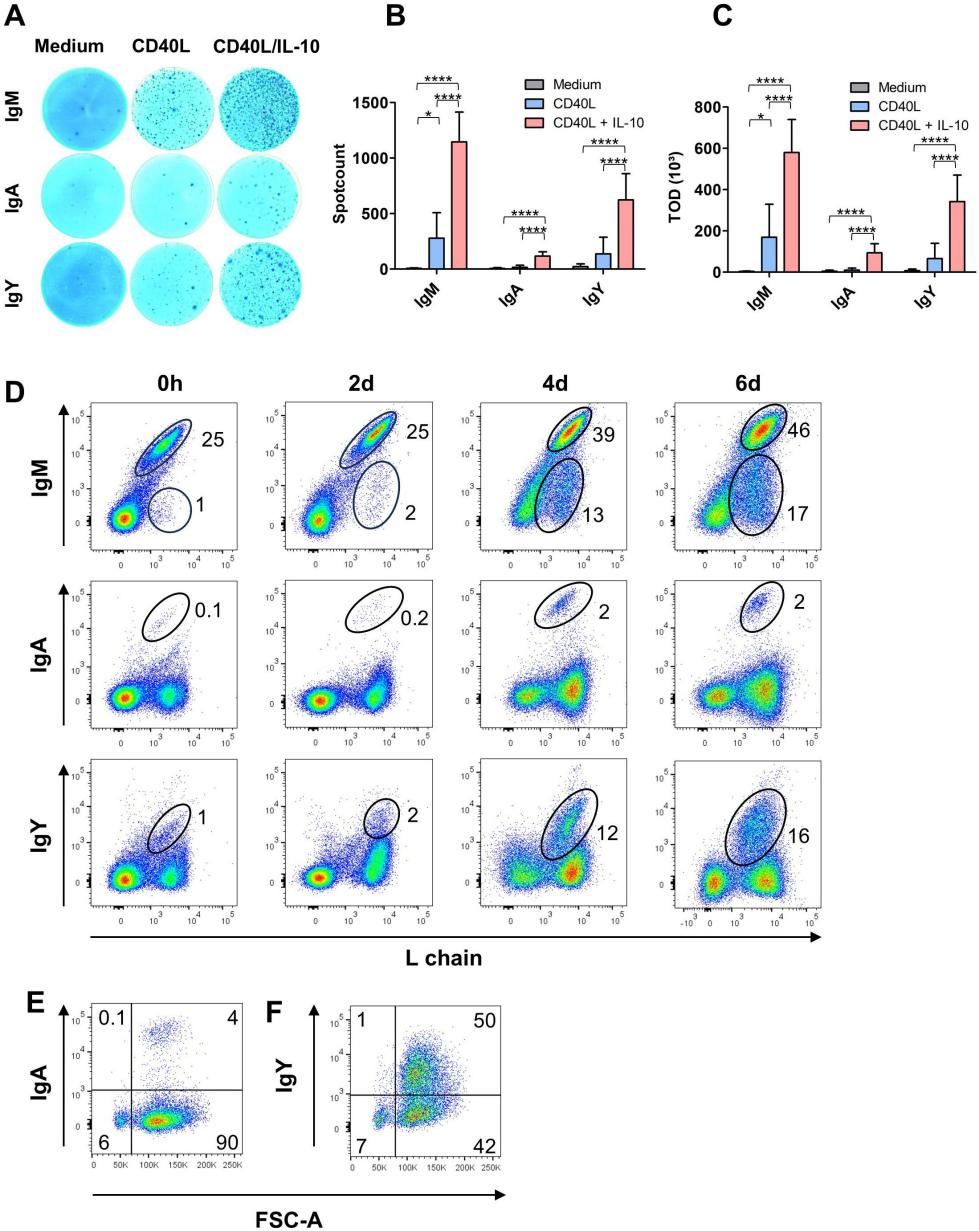
CD40L/IL-10 stimulation induces Ig secretion and class switch. **(A–C)** ELISpot-assays for total IgM, IgA and IgY from splenic leukocytes after six days of culture in the presence of the respective cytokines. **(A)** Representative photos from an ELISpot plate, **(B)** spot count **(B)** and **(C)** total optical density (TOD): Shown are mean ± SD of three independent experiments with *: P<0.5; ****: P<0.0001 by one-way ANOVA with Bonferroni *post hoc* test. **(D)** Surface expression for IgM, IgA and IgY was determined by flow cytometry on freshly isolated and CD40L/IL-10 stimulated cells after the indicated times of culture. Blots are gated for viable, single leukocytes. **(E, F)** Size (FSC) of class-switched cells after six days of CD40L/IL-10 stimulation. Blots are gated on L chain^+^, viable, single leukocytes. **(D–F)** Blots are representative for three independent experiments.

To analyze Ig production in CD40L/IL-10 stimulated cultures, splenocytes were stimulated for 6 days with CD40L or CD40L/IL-10 and antibody production was measured via ELISpot assay (**Figure 3A**).

Hardly any Ig producing cells were detectable in media controls, but the addition of CD40L resulted in clearly visible spots for IgM and IgY while only very few IgA^+^ cells were found. The combination of CD40L and IL-10 had a strong synergistic effect on the secretion of all three Ig isotypes and led to a 4fold higher number of IgM^+^ spots and a 5fold increase of IgY^+^ spots and a 7fold increase of IgA^+^ spots (**Figure 3B**). The strong synergism of CD40L/IL-10 was also observed for the total optical density (TOD), the integral accumulation of area and intensity of each spot, which next to the number of antibody secreting cells also includes the secreted amount of antibodies (**Figure 3C**).

As the ELISpots had revealed the presence of class-switched, antibody secreting cells in the CD40L/IL-10 stimulated cultures, we examined whether this was also linked to changes in BCR surface expression. When cells were analyzed for Ig surface staining by flow cytometry at different timepoints, IgM remained the dominant surface immunoglobulin over the stimulation period of 6 days (93, 75, 73% of the B cells at days 2, 4 and 6, respectively). But in addition to IgM, a small but clearly defined population of IgA^+^ cells was detectable after 4 and 6 days representing 4% of L chain positive cells. And the culture system worked even better for class switch to IgY, as after 4 days about one quarter of the B cells in culture expressed an IgY type BCR (**Figure 3D**). Importantly, class switch occurred only in the blasting cells of the cultures, the remaining small B cells maintained a BCR of IgM isotype (**Figures 3E, F**).

### 3.3 Phenotype of *in vitro* generated plasma blasts

**Figure 4:**
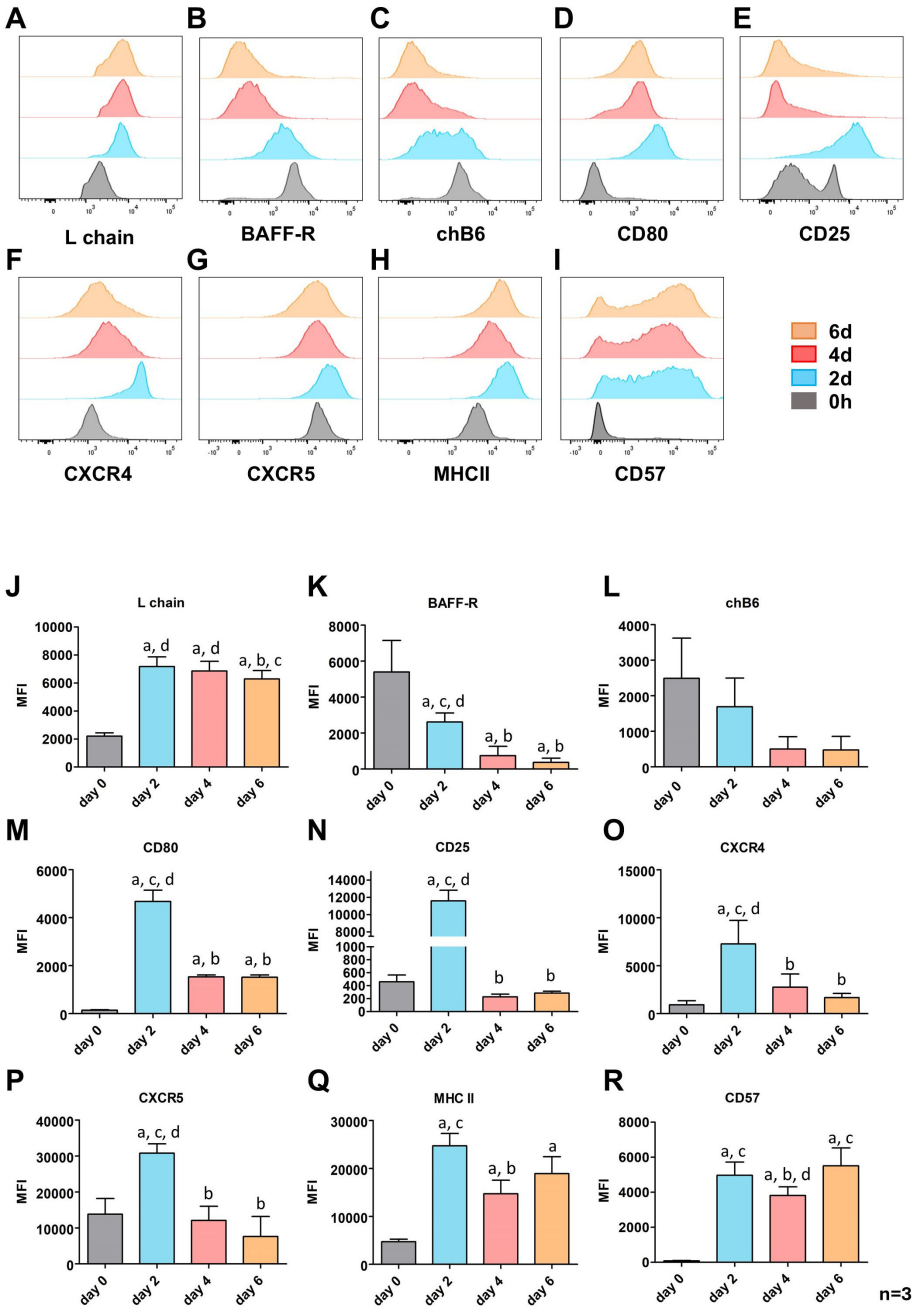
CD40L/IL-10 induced expression of surface markers in prolonged cultures. Splenic leukocytes were stimulated with CD40L/IL-10 for six days and analyzed for the expression of the indicated surface markers by flow cytometry analysis every second day. Populations are gated for L chain^+^, viable, single leukocytes at day 0 and proliferating (large), L chain^+^, viable, single leukocytes for the subsequent time points. **(A–I)** Surface marker expressions for one representative out of three experiments. **(J–R)** Mean ± SD of the mean fluorescence intensity (MFI; median) for three independent experiments. Statistics were calculated by two-way ANOVA with Bonferroni *post hoc* test. Letters indicate significant differences with p: ≤ 0.05 to day 0 (a), 2 (b), 4 (c) or 6 (d) of culture.

Having established a culture system that induces B cell proliferation, class switching, and antibody secretion, we next conducted flow cytometric phenotyping of the differentiating B cells. Analysis after 2 days had already shown that L chain expression remained high after CD40L/IL-10 stimulation. More prolonged examination revealed a constantly increased amount of surface BCR after 4 and 6 days, though the MFI for L chain staining on day 6 was slightly lower (**Figures 4A, J**). Thus, L chain staining picks up all B cells in stimulated cultures and for further phenotyping of B cell blasts, all samples were gated for large L chain positive cells.

The initially observed downregulation of BAFF-R expression continued stepwise after 4 and 6 days, resulting in an almost complete loss of this cytokine receptor at day 6 (**Figures 4B, K**). We also detected a further loss of chB6 within the proliferating cells, but in contrast to BAFF-R after 2 or 4 days two subpopulations became detectable, one with reduced chB6 expression and a smaller one which became nearly chB6 negative (**Figures 4C, L**). Hence, only a subpopulation of the cells can be considered as marker-negative/Ig positive cells. The variability between individual animals in the timing of the appearance of the two populations and their size resulted in a lack of statistical significance (**Figure 4L**).

The activation markers CD80 and CD25 showed a similar staining pattern, with a strong upregulation after 2 days followed by a strong drop at day 4 and 6. However, they differ in their initial expression as freshly isolated B cells are all CD80 negative while approximately one quarter of the cells show a medium CD25 expression. While CD25 expression returned to baseline levels at 0 hours, CD80 expression remained slightly and significantly elevated (**Figures 4D, E, M, N**).

The pattern of increased marker expression after two days followed by a downregulation at later timepoints was in general also observed for the chemokine receptors CXCR4 and CXCR5. They differ only in that CXCR4 was hardly found on freshly isolated cells, whereas all B cells from the spleen were CXCR5 positive (**Figures 4F, G, O, P**).

MHC class II expression, as indicator of the antigen presenting capacity of the cells showed an about 5fold increased expression at day 2 (**Figures 4H, Q**) and stayed significantly increased over time. Interestingly, in most animals we found a transient reduction in MHCII expression on day 4 followed by a renewed increase on day 6.

**Figure 5:**
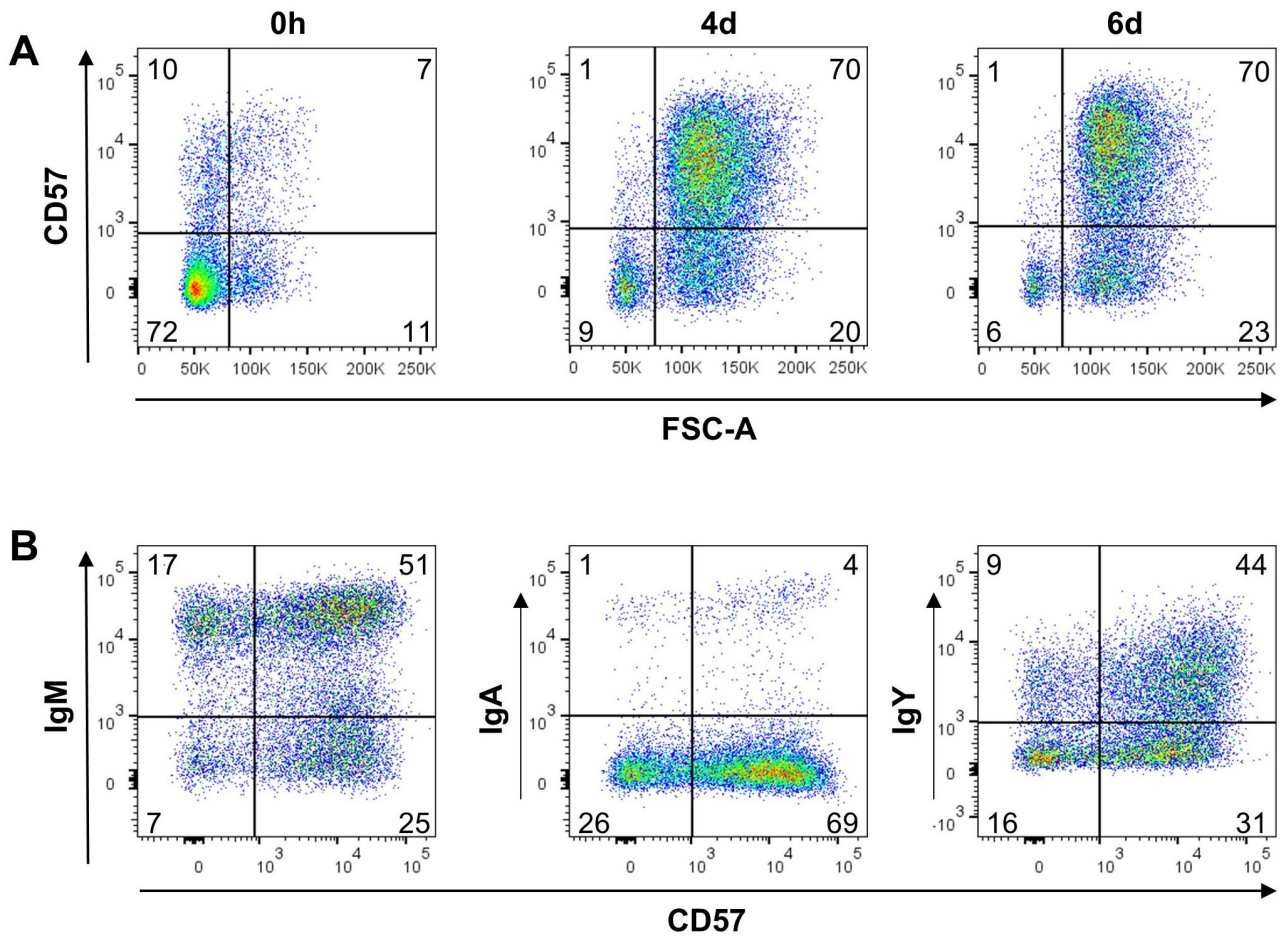
Increased CD57 expression is restricted to blasting cells in stimulated splenocytes. Splenocytes were stimulated with CD40L/IL-10 and analyzed for CD57 expression by flow cytometry. Shown data are representative for three independent experiments. **(A)** Size (FSC) of CD57 expressing L chain^+^ cells at the indicated timepoints; blots are gated for L chain^+^, viable, single leukocytes. **(B)** CD57 expression on class-switched cells after six days of culture; blots are gated on blasting, L chain^+^, viable, single leukocytes.

CD57 has been proposed as a possible plasma cell marker in chickens (56). Only a very small fraction of freshly isolated splenic B cells expresses CD57, but CD40L/IL-10 stimulation induced a 60fold upregulation of CD57 on day 2, and only a small proportion of cells remains negative. This pattern remained largely unchanged at day four and six of stimulation, though in parallel to MHCII, on day 4 a transient reduction of CD57 from a median MFI of about 5000 below 4000 could be observed (**Figures 4I, R**). Gating for small and blasting Ig^+^ cells revealed that CD57 upregulation is largely restricted to blasting cells (**Figure 5A**). Relating CD57 and Ig surface expression, showed that IgA^+^ and IgY^+^ class-switched cells can be found both in the CD57^+^ and CD57^-^ fraction (**Figure 5B**). Hence, CD57 expression accompanies blast formation but CD57 expression and class switch seem to occur independently.

In summary, CD40L/IL-10 stimulated B cells retain BCR positivity, exhibit a continuous loss of BAFF-R and chB6 expression, display a moderate upregulation of MHC class II, and show a marked upregulation of CD57. For the activation markers and chemokine receptors analyzed, an initial activation peak at day 2 is followed by a subsequent downregulation of these markers, clearly delineating an early activation phase from later time points in the culture.

### 3.4 Transient expression of TACI and CD138 during *in vitro* B cell differentiation

Though phenotyping of CD40L/IL-10 differentiated B cells has revealed interesting staining patterns, no specific marker for those cells was identified among the available mAbs. As human plasma cells can be addressed by CD138 expression (13, 14) and both memory B and plasma cells show a switch from BAFF-R to TACI expression, we generated new chicken specific antibodies for these molecules (Staining on transfected cells is shown in **Supplementary Figure 4**).

**Figure 6:**
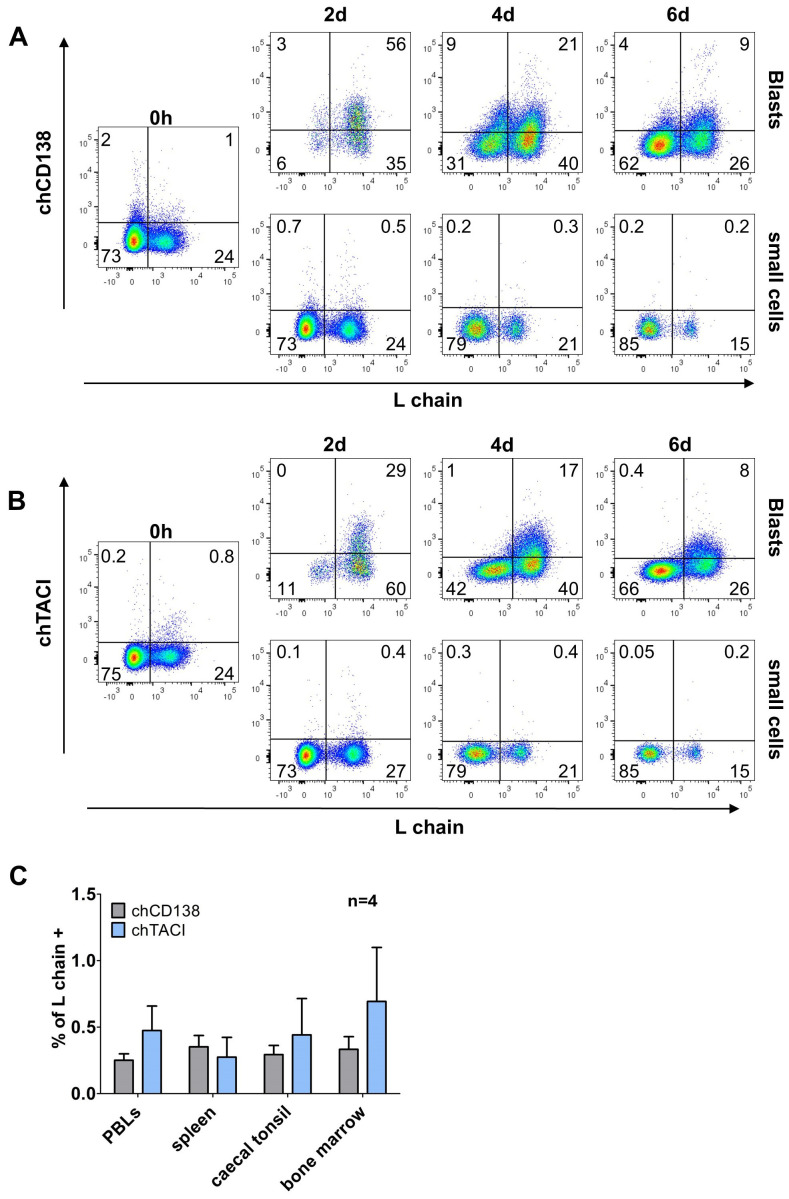
Time-limited expression of chCD138 and chTACI on activated B cells in splenocyte cultures. **(A, B)** Splenocytes were stimulated with CD40L/IL-10 and analyzed by flow cytometry for expression of chTACI **(A)** or chCD138 **(B)** at the indicated timepoints. Blots are gated for viable, single leukocytes and either large, blasting cells or small cells are shown. Data are representative of three independent experiments. **(C)** Expression of CD138 and TACI on L chain^+^ cells from different lymphoid organs. Flow cytometry blots were gated for viable, single CD45^+^/L chain^+^ leukocytes). Shown are mean ± SD of four independent experiments.

Staining of CD40L/IL-10 stimulated cells revealed that freshly isolated cells are vastly negative for both markers. However, after two days in the presence of CD40L/IL-10 two thirds of the blasting B cells expressed CD138 and one third expressed TACI (**Figures 6A, B**). However, distinct expression of both markers declined at later timepoints.

Analysis of light chain positive, proliferating cells revealed that at day two, nearly all cells that were positive for chTACI also expressed chCD138 but the majority of chCD138 positive cells remained negative for chTACI (**Supplementary Figure 7A**). As BAFF-R and TACI are both receptors for the cytokine BAFF, we examined the co-expression of TACI and BAFF-R, on light chain positive proliferating cells. After two days, all TACI-expressing cells were also BAFF-R^+^. But at later timepoints, when expression of both receptors decreased, a small proportion of the cells still expressed a low amount of BAFF-R, but the majority of light chain positive cells expressing neither of both BAFF receptors (**Supplementary Figure 7B**).

Among freshly isolated leukocytes from different lymphoid tissues below one percent of the cells was positive for CD138 and TACI. The majority of positive cells were L chain negative/non-B cells. The only exception were caecal tonsil leukocytes, where the few CD138^+^ and TACI^+^ cells were L chain^+^ (**Figures 6C**).

Hence, neither CD138 nor TACI seem to be expressed throughout the differentiation process to plasma cells but rather at a short time window after initial cell activation.

### 3.5 Distribution of CD57 expression on primary B cells

**Figure 7:**
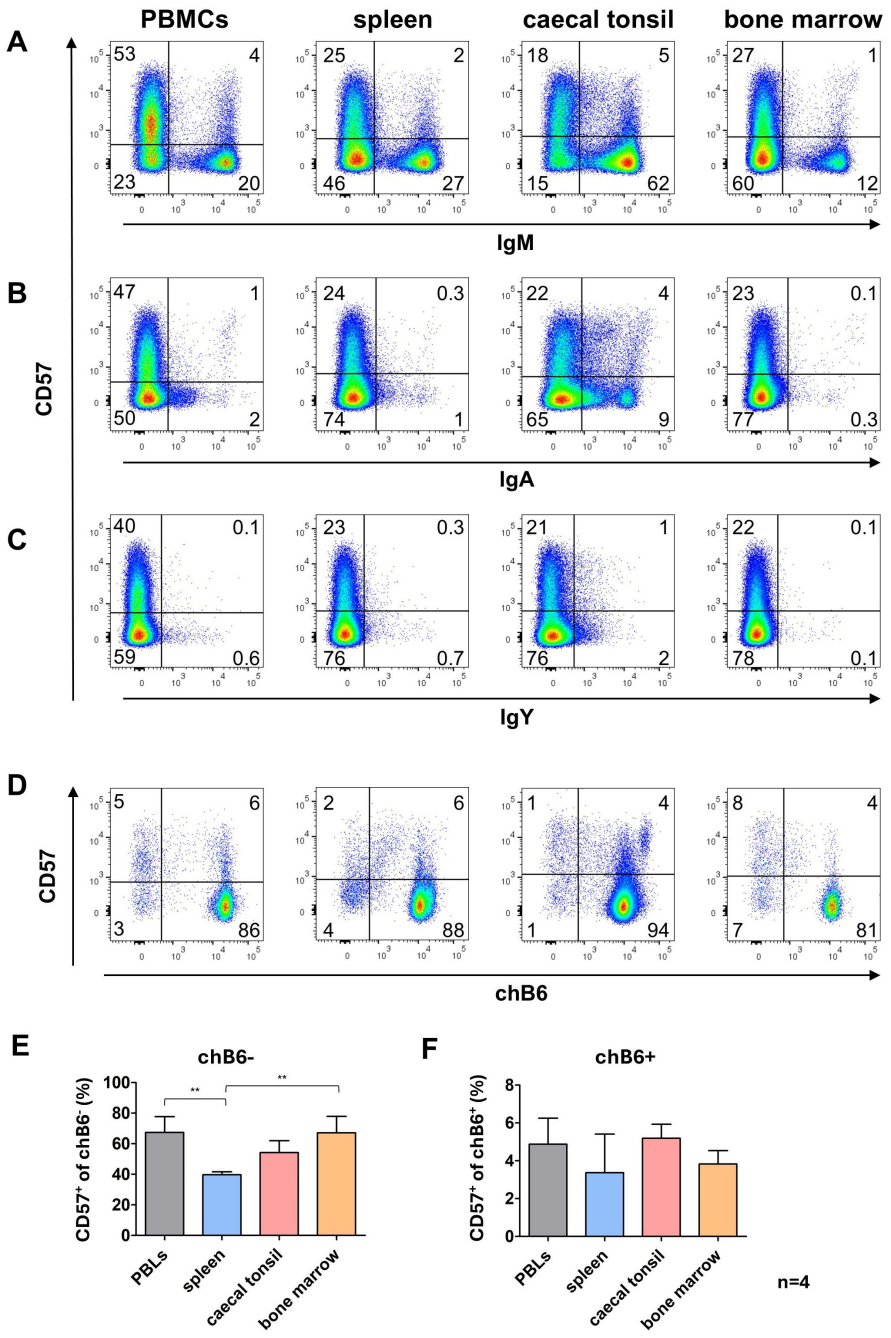
CD57 expression in lymphoid tissues. Leukocytes were isolated from the indicated tissues and analyzed for CD57 expression by flow cytometry. **(A–C)** CD57 expression on IgM^+^ and Ig class-switched cells, with blots gated for viable, single leukocytes. **(D)** CD57 expression on marker-negative (chB6^-^) and marker-positive (chB6^+^) L chain positive leukocytes. Blots are gated for viable, single, CD45^+^ cells. Shown are representative blots of four independent experiments. **(E, F)** Proportion of CD57^+^ cells among chB6^-^/L chain^+^ **(E)** and chB6^+^/L chain^+^ cells **(F)**. Statistics were calculated by one-way ANOVA with Bonferroni *post hoc* test with **: p ≤ 0.01, n=4.

Since the *in vitro* system had revealed an interesting staining pattern for CD57 on proliferating and class-switched cells, we investigated CD57 expression on primary B cells from different lymphoid tissues. Only a fraction of B cells from all analyzed organs was CD57^+^. The lowest proportion of CD57^+^ cells was found among unswitched IgM^+^ cells ranging from 4% in spleen up to 7% in the blood. A much higher percentage of CD57^+^ cells was detected in cells post class switch to IgA with about 30% of IgA^+^ cells in blood, CTs and bone morrow expressing CD57 while around 20% of IgA^+^ blood B cells expressed CD57. Due to the detection of only a few IgY^+^ cells, clear evidence is lacking; however, it appears that the majority of IgY^+^ cells do not express CD57 (**Figures 7A–C**).

Relating CD57 expression on L chain positive cells to chB6 downregulation demonstrated that in all organs much more marker-negative cells expressed CD57 (40-67%) compared to the marker-positive fraction (3-5%) (**Figures 7D–F**). This could be indicative of a higher activation level of these cells, but our data do not show that CD57 expression is indicative of a defined B cell differentiation stage as memory or plasma cells.

### 3.6 Influence of age and vaccination

To analyze the influence of bursa involution and frequent immune responses over time, we determined the frequency of class-switched cells in blood and spleen of commercial layers at the age of seven weeks (fully developed bursa), 17 weeks (beginning bursa involution, before the start of laying), 27 weeks (no bursa, laying) and 70 weeks (end of laying, before moult).

**Figure 8:**
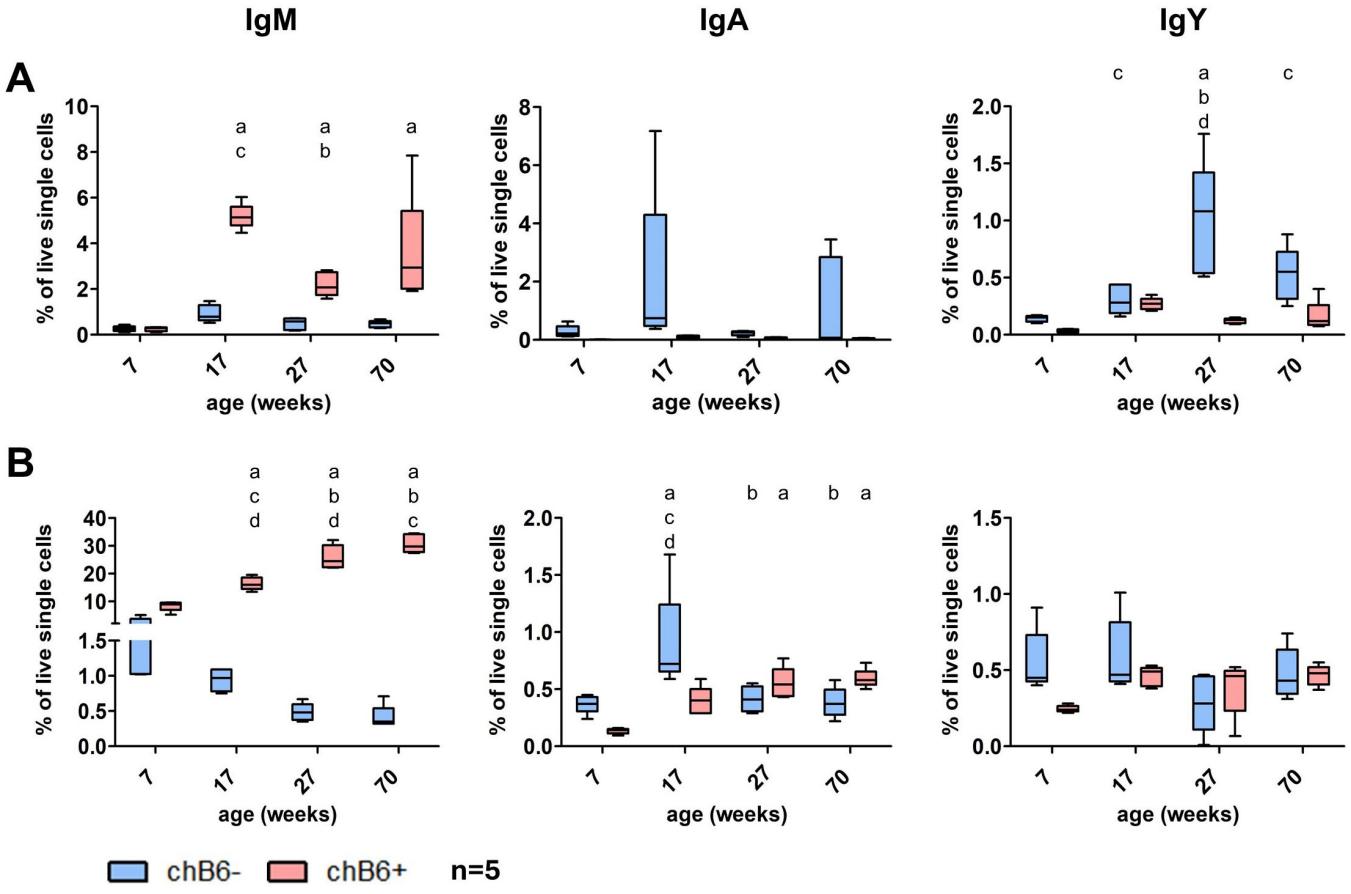
Impact of age on class-switched cells. Leukocytes were isolated from blood **(A)** and spleen **(B)** from chickens of different age and examined for sIg expression on chB6^-^ and chB6^+^ cells by flow cytometry. Frequencies were obtained from viable, single leukocytes; n=5 per group; whiskers show minimum to maximum; Statistics were calculated by two-way ANOVA with Bonferroni *post hoc* test. Small letters display significant differences with p: ≤ 0.05 to week 7(a), 17(b), 27(c) and 70(d).

Overall, in these birds, percentages of both chB6^+^ and chB6^-^ BCR positive cells in the blood were quite low, but we detected more chB6^-^ IgA and IgY positive cells (the potentially class-switched plasma cells) than unswitched chB6^-^/IgM^+^ cells. Highest percentages for both chB6^-^ and chB6^+^ IgM^+^ and IgA^+^ cells and chB6^+^/IgY^+^cells were detected at 17 weeks just before laying. Only chB6^-^/IgY^+^ cells peaked later at 27 weeks (**Figure 8A**). The splenic B cell compartment was characterized by a strong and continuous increase in chB6^+^/IgM^+^ cells over time. For all other populations only very low and quite constant frequencies where detected (**Figure 8B**). Hence, we could not detect a distinct increase of class-switched cells over time, only the begin of laying seems to have some effect.

**Figure 9:**
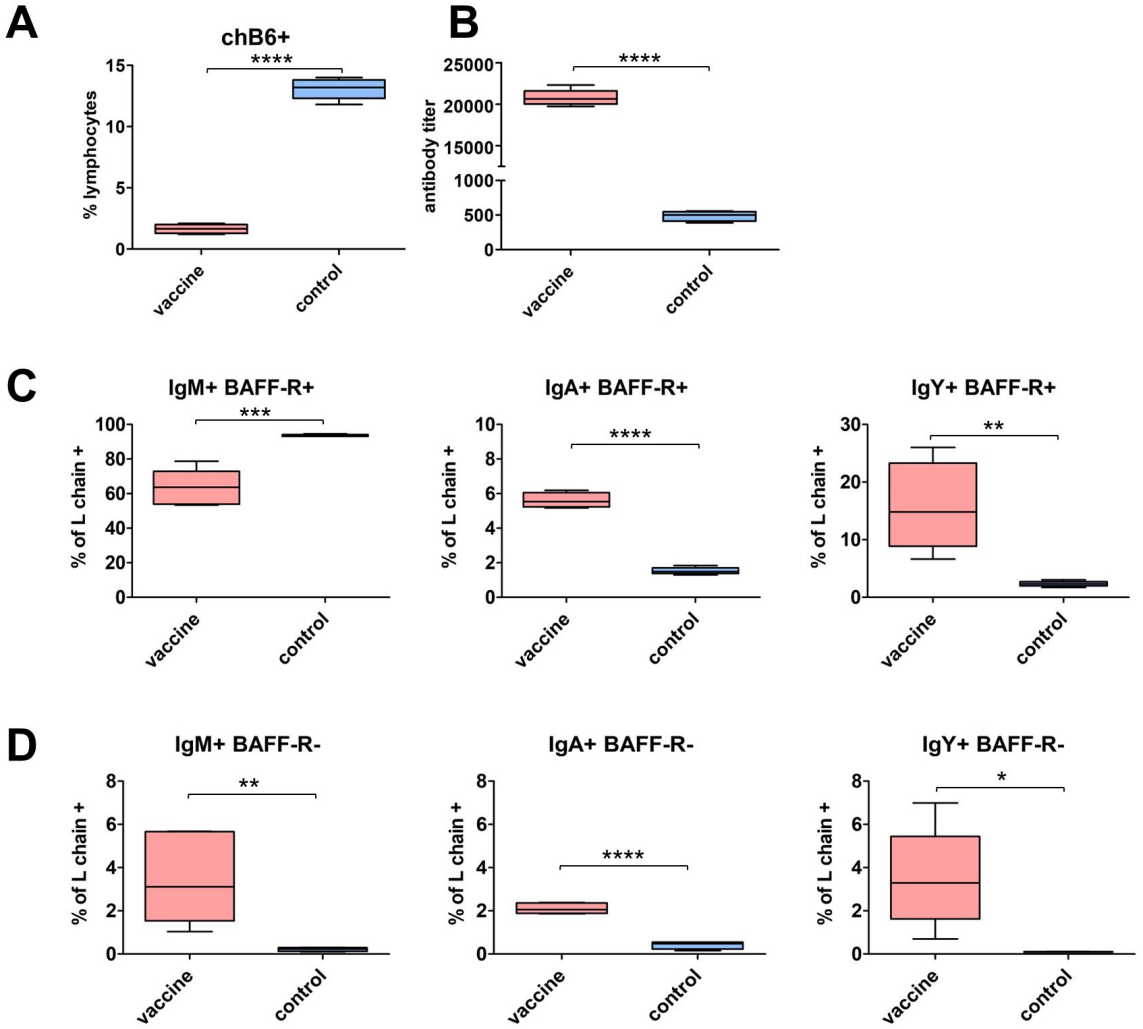
Impact of IBDV vaccination on class-switched cells. Six week old birds were vaccinated with a IBDV live vaccine and blood samples were analyzed 28 days post vaccination in comparison to unvaccinated controls. **(A)** Frequency of B cells (chB6^+^); **(B)** IBDV specific IgY antibody titer in sera were quantified by ELISA. **(C, D)** Percentage of Ig surface expressing cells from marker-positive (L chain^+^/BAFF-R^+^) **(C)** and marker-negative (L chain^+^/BAFF-R^-^) **(D)** B cells. n=5 in vaccinated group and n=3 in control group; whiskers show minimum to maximum, Frequencies were obtained from viable, single CD45^+^ lymphocytes **(A)** and L chain^+^ viable, single CD45^+^ lymphocytes **(C, D)**. **(A–D)** *: p ≤ 0.05; **: p ≤ 0.01; ***: p ≤ 0.001; ****: p ≤ 0.0001 by unpaired t-test.

Finally, we assessed whether we could detect an influence of vaccination on the presence of class-switched cells. Therefore, chickens were vaccinated with an intermediate virulent IBDV vaccine strain. This vaccine is reported to induce acute B cell death in the bursa in the first week p.v. but also high IBDV specific antibody titers (57). We found that at day 28 p.v. the peripheral B cell system is recovering, though the fraction of peripheral B cells is still reduced (**Figure 9A**). We measured homogenously high antibody titers in all birds and as the ELISA system detects IgY we could also demonstrate class switch (**Figure 9B**). When we analyzed Ig expressing cells in the blood, we detected a significantly lower percentage of IgM^+^/BAFF-R^+^ cells in vaccinated birds, but all other chB6^+^ and chB6^-^ Ig^+^ populations showed a highly significant increase in vaccinated birds (**Figures 9C, D**). Thus, IBDV vaccination has led to a substantial increase in chB6^-^ cells and a significantly increased fraction of class-switched cells.

## 4 Discussion

In chickens little is known about class switch, affinity maturation, live time and location of plasma and memory cells, but a better understanding of these processes is necessary to develop more precise and specific vaccination strategies.

Due to the absence of specific differentiation markers for chicken B cells like CD19, CD20, CD27, CD38 and CD138, our approach was to discriminate naïve from differentiated B cells by identifying cells that have undergone Ig class switching. Overall, we found relatively few IgY and IgA positive cells in the analyzed tissues with frequencies from 1% to 2% of B cells in blood, spleen and BM. For humans it was shown that about 20 to 25% of blood B cells are class-switched cells (38, 58, 59), the majority of them representing memory cells (60). Hence, the frequencies observed for the chicken were substantially lower.

Only in the caecal tonsils, which are gut-associated secondary lymphoid structures rich in GCs, we identified a significant population of IgA^+^ class-switched cells (7). This had already been shown by immunohistochemistry (61, 62), now we demonstrate that it is also possible to detect class switch to IgA by surface staining for flow cytometry. We could not detect a significant number of IgY-positive cells in any of the tissues examined, including the bone marrow, where in mammals long lived IgG^+^ plasma cells (63–65) reside in specific survival niches. This is in accordance with observations from De Geus and colleagues, who by ELISpot also detected no differences in the number of IgY secreting cells between blood, spleen and bone marrow (66). However, given the very low frequency of plasma cells in human bone marrow preparations, which is reported to be below 0.5% (67, 68) our observations do not exclude the possibility of similar survival niches for IgY plasma cells in the chicken bone marrow.

### 4.1 B cell differentiation *in vitro*

To enable the detailed characterization of a larger number of cells, we aimed to establish a culture system for chicken B cells that supports B cell differentiation and immunoglobulin class switching. We had already shown that trimerized soluble CD40L induces strong proliferation and long-term survival of chicken B cells (23). Based on reports that the addition of IL-10 to CD40 activated human B cells induced antibody secretion and class switch to IgG (50, 69) and the description of a functional chicken IL-10-homologue (51), we combined CD40L and IL-10 in cultures of mixed chicken spleen leukocytes. We found a strong synergistic effect of CD40L and IL-10 on cell proliferation, which in the first days of culture was almost completely composed of B cells. Transcription factor analysis after two and four days of culture revealed very different profiles in small and blasting cells and demonstrated differentiation of the blasting cells toward plasma cells with a strong decrease in *Pax5* and increase in *Blimp1* expression (70). This was also supported by the upregulation of *µS* (the soluble form of IgM) (71). These ASCs were indeed detected in ELISpot assays with highly significant increases of spot numbers of all Ig isotypes. Compared to the Ig isotype distribution in freshly isolated cells, CD40L/IL-10 stimulation clearly changed the ratio toward isotype-switched cells with almost as many IgY and IgA ASCs than IgM secreting cells. In addition, we found strongly increased numbers of class-switched cells to both IgA and IgY by flow cytometric staining. This increase could be caused either by increased survival of the few class-switched cells in the starting population or the induction of class switch recombination (CSR). Supportive of the latter is the found upregulation of *AICDA*, the essential enzyme for CSR (72, 73), that we observed in both small cells and blasts, and which was also reported for equally activated human B cells (50).

Thus, by stimulating splenic leukocytes with CD40L and IL-10, we established culture conditions leading to B cell proliferation, antibody secretion and an increased number of isotype-switched, preferentially IgY^+^, cells, which provide enough cells for a more detailed phenotypic analysis.

### 4.2 Phenotype of activated B cells

Surface staining for L chain expression on CD40L/IL-10 activated B cell blasts showed that development of ASCs did not induce BCR downregulation for any Ig isotype. This contrasts the long-time dogma for mammalian plasma cell differentiation, that cells switch from membrane-bound to secreted Ig expression (74–76), resulting in the loss of surface BCR expression. However, more recent data show an isotype dependent BCR loss. While IgG expressing plasma cells lose membrane bound Ig during their differentiation toward long lived plasma cells (77), IgM and IgA expressing plasma cells keep a functional BCR on their surface (77, 78). *In vitro* generation of plasma cells from human memory cells resulted in a similar picture with a reduction of BCR expression for IgM and IgA and a complete switch to intracellular Ig for IgG expressing cells (79). Our cultures were not extended beyond 6 days and culture conditions were not optimized for a terminal differentiation, which would require the withdrawal of CD40L (46, 79). Therefore, we cannot rule out that longer time of culture and different conditions could lead to BCR downregulation on chicken plasma cells. However, at least for this first phase of activation and ASC induction membrane expression of all BCR isotypes remained high, suggesting a different regulatory mechanism for IgY compared to IgG expression.

Beside the possible functional implications of this observation, like the continuous ability of these cells for antigen recognition, prolonged BCR expression was also of technical importance. In the absence of specific markers, it allowed us to gate the mixed leukocyte cultures for L chain^+^ cells for a detailed phenotyping of differentiating B cells.

Like the BCR, MHC class II expression increased rapidly and remained at a very high level, demonstrating that B cells generated in our system keep both their capability to recognize antigen by the BCR and present antigen to T helper cells by MHC class II. Human B cells retain MHC class II expression in the GC and on memory and short-lived plasma cells and lose it only when differentiating into long-lived plasma cells (80), suggesting that this final differentiation step is not induced in our present system.

We found a reduced abundance of chB6 mRNA and a continuous reduction of surface expression from cell isolation to day six of CD40L/IL-10 stimulated cultures. This is in line with downregulation of chB6 on CD40L induced plasmablasts (23) and lack of chB6 staining on plasma cells detected by less-sensitive immunohistochemistry (21) and collectively suggests that chB6 expression is lost during the final differentiation of chicken B cells.

Interestingly, after two to four days of CD40L/IL-10 activation, chB6 expression became biphasic, with only part of the L chain^+^ blasts showing a complete loss. This can either argue for an ongoing process toward a complete loss or indicates differentiation of different subpopulations and may represent a plasma and memory cell pathway, though *BCL-6*, the memory cell associated transcription factor was strongly upregulated in small cells.

BAFF-R, exclusively expressed on B cells and one of the chicken’s two receptors for the B cell survival cytokine BAFF (25) was gradually and almost completely lost over the time of culture. In human B cell differentiation, only the plasma cell pathway (plasmablasts, short- and long-lived plasma cells) loses BAFF-R (81), while memory cells remain BAFF-R positive (82). This would argue against a possible memory B cell development in CD40L/IL-10 stimulated cultures. However, early prebursal and bursal chicken B cells (26) express BAFF-R, which is in contrast to BAFF-R expression only from the transitional B cell stage on in mammalian B cells (83, 84). This demonstrates significant differences in the expression profile of BAFF receptors between avian and mammalian B cells, which could also account for memory B cells.

Both, chB6 and BAFF-R are B cell markers which are expressed throughout chicken B cell development from the prebursal B cell stage onwards (85, 86). Their loss on differentiating culture cells along with consistent Ig surface expression makes it very likely that the observed population of BAFF-R^-^/chB6^-^ (marker-negative) and Ig^+^ cells, which we found *ex vivo*, represents further differentiated B cell stages and could contain plasma cells. But while in cultures a gradual loss of chB6 and BAFF-R was visible, cells in blood and tissues cells were mostly either marker-positive or marker-negative, with only few Ig^+^ cells in spleen and CT showing an intermediate chB6 expression.

About one third of freshly isolated spleen B cells expressed CD25, the high-affinity chain of the IL-2 receptor. Among human blood B cells, the majority of memory B cells expresses CD25, while only 10 to 20% of naïve B cells and ASCs are CD25^+^ (87). Another report describes the number of CD25^+^ B cells in the blood with 2% and 7% in the spleen (88). Hence, the detected number of CD25^+^ B cells in chickens was substantially higher and strongly enhanced by CD40L/IL-10 activation in the first days of culture. Interestingly, it vanished almost completely at later timepoints, highlighting that i) despite the continuous presence of both CD40L and IL-10 two distinct phases of activation and differentiation exist in these cultures and ii) either no memory cells are induced in these cultures or chicken memory cells do not express CD25.

CD80 expression also peaked on day two but lower levels of CD80 expression persisted at later time points. For human and murine B cells a strong upregulation of CD80 expression is associated with GC B cells (89, 90), it is partially retained on memory cells but lost during the differentiation of plasma cells (91). On the assumption that expression during chicken B cell differentiation is regulated similarly, that would imply an ongoing GC like differentiation process in our culture system.

The observed initial CXCR4 upregulation with a subsequent rapid loss of its expression is also in favor of a GC-like activity in our culture system. Proliferating mammalian GC B cells upregulate CXCR4 to retain them in the CXCL12 high dark zone of the GC (92–94). Upon their final differentiation, memory and plasma cells then downregulate CXCR4, to allow them to leave the GC.

### 4.3 Applicability of CD57, CD138 and TACI

In mammals, CD57 is used as marker for cells of neural crest origin (95–97), and different NK (98, 99) and T cell subpopulations (100) (101). CD57 is a terminally sulfated carbohydrate epitope (glucuronic acid 3-sulfate) (102–104), but to date little is known on which molecules the CD57 epitope is actually expressed. Our analysis of chicken leukocytes from different tissues showed that the majority of non-B cells are CD57^+^. Since CD57 was reported as activation marker for chicken T cells (105), this suggests a similar expression pattern for mammalian and chicken T cells. In contrast, CD57 expression on human B cells is a rather rare event and only few B cells in the blood are CD57^+^ (106), though a higher CD57 expression on B cells has been shown in patients with relapsing-remitting Multiple Sclerosis (106).

We investigated CD57 expression, as it was described as marker of chicken B cell activation and plasma cells (56) and could indeed detect a continuous upregulation of CD57 on most blasting cells post CD40L/IL-10 activation. Interestingly, throughout the whole culture period, a quarter of B cell blasts remained negative for CD57 and its expression was independent of Ig class switch. This distinguishes CD57 from other activation markers like CD25 and CD80, which were homogenously up- and downregulated on all blasts. In freshly isolated leukocytes from different tissues, only a small fraction of B cells was CD57^+^. However, more class-switched IgA^+^ cells (up to 30%) expressed CD57 than unswitched cells and tissue dependent, more marker-negative/Ig^+^ cells (up to 60%) than marker-positive/Ig^+^ cells, which indicates a higher activation state in these subpopulations.

Analyzing CD57/chB6 expression by flow cytometry, Mast and colleagues identified three different B cell populations in spleen and cecal tonsil, describing them as CD57^-^/chB6^high^ resting B cells, CD57^+^/chB6^high^ GC B cells and CD57^+^/chB6^low^ plasma cells (56). This classification was based the observation that immunohistochemistry showed weak CD57 staining in GCs and a lack of CD57 on B cells in the periellipsoidal white pulp. However, this interpretation of flow data is not supported by data. At least in the spleen, standard leukocyte preparations do not contain GC B cells as the encapsulated GCs stick to the artery tree (107) and are not included by regular isolation protocols. In addition, some chB6 expression can also be found on macrophages (108) but no further characteristics of the CD57^+^/chB6^low^ population were described. Though we also found these populations, we think designation of these two populations as GC and plasma cells seems not really supported.

The observed, frequent CD57 expression on chicken B cells is in contrast to mammalian data and no parallels can be drawn from human and mouse B cells. In chickens, CD57 can be considered as a marker for a specific kind of B cell activation. Interestingly, bursal B cells, which proliferate and undergo BCR diversification, are all CD57^+^, while the majority of blood B cells are negative. Hence, it seems justified to consider CD57^+^ B cells as non-resting cells undergoing differentiation, though we currently cannot link its expression to a specific stage of B cell differentiation like GC, memory or plasma cells.

With the intent of getting plasma/memory cell specific markers, we generated two new mAbs directed against the chicken homologues of CD138 and TACI. Even though CD40L/IL-10 stimulation induced expression of TACI and CD138 on blasting cells, both molecules were downregulated post day two and only a very weak surface staining remained. Hence, in contrast to mice and humans (14, 16, 109) neither of them seems to be a marker for further differentiated B cells but rather indicates a form of B cells activation, like the processes which occur in GCs. This could also be an explanation for the very low frequency of CD138 or TACI positive cells in spleen and cecal tonsil as GC B cells are probably absent (spleen) or present in unknown numbers (CT). As only a fraction of B cell blasts becomes clearly CD138 and TACI positive, it will be interesting for future studies to characterize these positive and negative cells in more detail.

### 4.4 Improvement of the biphasic culture system

Surface marker and transcription factor analysis of differentiating B cells in our culture system revealed a biphasic process in CD40L/IL-10 stimulated mixed cultures of splenic leukocytes. We see an initial phase of cell activation (e.g. upregulation of CD25, CD80, TACI, CD138) followed by a second phase of increased cell differentiation (high *BLIMP-1* expression, loss of chB6 and BAFF-R, class switch). As mentioned above, this system was not optimized for final B cell differentiation. For cultures of both naïve and human GC B cells it was shown that termination of CD40L co-stimulation stops proliferation and promotes differentiation into antibody-secreting cells (110), while the continued presence of CD40L in GC cultures induced memory cells (46). Hence, in the future the removal of CD40L from the culture could help to induce further differentiation. In analogy to processes in mammalian GCs, addition of other cytokines could be necessary to generate more defined B cell phenotypes. A promising candidate is IL-21, which in mammalian GCs is produced by follicular T helper cells and strongly increases the percentage of CD38^high^/CD138^high^ plasma cells as well as antibody production of CD40L activated human B cells (111). We cannot rule out that some IL-21 was already present in our mixed cultures as the large amount of proliferating T helper cells detected at later timepoints could be a source of IL-21.

Another cytokine, which is often included in mammalian B cell cultures is IL-2 (46, 112). The strong and rapid upregulation of CD25, the IL-2 receptor alpha chain, on CD40L/IL-10 stimulated B cells suggests that these chicken B cells may also respond to IL-2 and possibly, IL-2 was already present in the cultures: The emergency of a large T helper cell population in CD40L/IL-10 stimulated splenocyte cultures is unlikely to be a direct effect of CD40L/IL-10, as IL-10 itself is known to suppress proliferation of T helper cells (113) instead of inducing it. However, it was shown that CD40 activated human and rabbit B cells can produce IL-2 (114, 115). If a similar reaction occurs in chicken B cells, this could be one possible explanation for the observed differentiation processes and cellular composition in our culture system. To identify the exact effects of individual cytokines, cultures of sort purified cells will be helpful in the future.

### 4.5 Age and antigen exposure

Though, we had not identified a specific marker for plasma or memory cells from analysis of *in vitro* stimulated B cells, the cultures had demonstrated that during the induced differentiation processes BCR surface expression, regardless of the Ig isotype, is not reduced and proven that BAFF-R and chB6 are lost during B cell differentiation. As all B cells, which emigrate from the bursa are chB6/BAFF-R positive, this allows the discrimination of marker-positive B cells from marker-negative B cells, which have undergone further differentiation. Based on these findings, we have examined the presence of marker-positive and marker-negative class switched cells under the influence of bursal involution and antigen exposure.

Loss of the bursa as primary B cell organ at the age of sexual maturity means a dramatic switch in the composition of the peripheral B cell pool. In birds with a productive bursa, about 60% of blood B cells are recent bursal emigrants with a half-life time of 30 hours (116), a population which misses post involution. In addition, frequent immunization and environmental antigen exposures add up over a chicken’s lifetime, hence one could expect an increase in class-switched cells over time.

Surprisingly, bursa involution does not reduce the frequency of B cells in blood and spleen. In contrast, IgM^+^ unswitched, marker^+^ cells increased in both blood and spleen. We observed an increase in and marker-negative cells around the beginning of bursa involution (17w; IgA^+^) or in the middle of the laying period (27w; IgY^+^). However, this was no continuous change until the end of the laying period (70w) and can more likely be attributed to the vaccination scheme before the beginning of the laying period than an increase due to lifetime. Interestingly, in human blood, plasma cells represent age dependent around 1-2% of circulating B cells (60), which fits very well with our data and supports the staining strategy.

Another strong intervention into the B cell system was the application of a live IBDV vaccine, leading to B cell depletion in the bursa, which is followed by recovery of the organ (117). At the same time, the vaccine induces a strong anti-IBDV antibody response, which we thought could be associated with an increase of marker-negative and class-switched B cells. Four weeks post vaccination the proportion of the different fractions had completely changed, though it has to be considered that the B cell numbers were still quite low. Both, marker-positive and marker-negative class-switched as well as marker-negative unswitched IgM^+^ cells, which are all correlates of differentiated cells, were significantly increased in vaccinated birds. This effect could be elicited either by an increased resistance of differentiated cells to IBDV infection or an increased generation of new IBDV specific cells. To address the causation, further studies with the determination of absolute cells counts and the identification of IBDV specific cells would be helpful. As memory cells should increase after vaccinations, the observed increase in class-switched cells in this first experiment showed that a more detailed comparison of marker-positive and negative class-switched cells post vaccination can be a very promising approach.

Overall, we have for the first time shown, that IL-10 together with CD40L induces strong activation of B cells in cultures of total chicken splenocytes, which includes proliferation, Ig class switch and increased antibody secretion. Characterization of these cells revealed an early GC (CD80, CD25, MHC class II high) and a later plasmablast-like (loss of chB6/BAFF-R, CD25 and CXCR4) phenotype. Importantly, we found that BCR surface expression remains high during the observed differentiation processes. Based on this, we discriminated *in vivo* two populations of class-switched cells: chB6^+^ and BAFF-R^+^ marker-positive and likely plasma cells containing chB6^-^ and BAFF-R^-^ marker-negative cells, whose presence was less affected by age than by vaccination.

These results can now serve as a starting point to identify further differentiation factors *in vitro*, perform next generation sequencing of the cells for the identification of specific memory and plasma cell markers and evaluate the effects of different vaccines on their presence to obtain a better understanding into the chicken’s antibody response.

## Data Availability

The original contributions presented in the study are included in the article/supplementary material. Further inquiries can be directed to the corresponding author.
