# Supplementary material for: Characterization of class-switched B cells in chickens
Source: Front Immunol. 2024 Nov 21;15:1484288. doi: 10.3389/fimmu.2024.1484288 (PMC11617357; doi:10.3389/fimmu.2024.1484288)
Supplement: Supplementary file 1 [file DataSheet1.docx]

Supplementary Material

# Supplementary Tables

## Supplementary Table 1

**Table S1.** Frequencies of sIg^+^ /BAFF-R^-^ and sIg^+^ /BAFF-R^+^ leukocytes from different lymphoid organs based on Figure 1 (mean ± SDM; n=5)

|  | **IgM** | | **IgA** | | **IgY** | |
| --- | --- | --- | --- | --- | --- | --- |
|  | ***BAFF-R -*** | ***BAFF-R +*** | ***BAFF-R -*** | ***BAFF-R +*** | ***BAFF-R -*** | ***BAFF-R +*** |
| **PBLs** | 0.20 *± 0.14* | 19.00 *± 4.55* | 0.20 *± 0.08* | 0.64 *± 0.37* | 0.09 *± 0.07* | 0.18 *± 0.10* |
| **Spleen** | 0.13 *± 0.05* | 32.18 *± 5.80* | 0.10 *± 0.07* | 0.48 *± 0.21* | 0.07 *± 0.06* | 0.15 *± 0.08* |
| **Caecal tonsil** | 0.11 *± 0.07* | 51 ± *9.90* | 0.48 *± 0.17* | 7.18 *± 0.89* | 0.07 *± 0.09* | 0.15 *± 0.05* |
| **Bone marrow** | 0.12 *± 0.06* | 19.25 *± 6.99* | 0.05 *± 0.04* | 0.33 *± 0.17* | 0.10 *± 0.07* | 0.06 *± 0.04* |

## Supplementary Table 2

**Table S2.** Leukocytes from different lymphoid organs were stained for CD57 and the respective surface Ig (IgM, IgA or IgY). Frequencies of the different subpopulations shown in Figure 7 (mean ± SD; n=5)

|  | **IgM** | | | **IgA** | | | **IgY** | | |  |
| --- | --- | --- | --- | --- | --- | --- | --- | --- | --- | --- |
|  | **CD57+/ Ig-** | **CD57+/Ig+** | **CD57-/Ig+** | **CD57+/ Ig-** | **CD57+/Ig+** | **CD57-/Ig+** | **CD57+ Ig-** | **CD57+/Ig+** | **CD57-/Ig+** |  |
| **PBL** | | 40,95 *± 5,7* | 1,49 *± 0,9* | 19,35 *± 4,9* | 41,45 *± 3,5* | 0,47 *± 0,1* | 0,96 *± 0,3* | 35,98 *± 3,7* | 0,32 *± 0,4* | 0,46 *± 0,1* |
| **spleen** | | 20,68 *± 4,8* | 1,35 *± 0,5* | 32,28 *± 5,8* | 23,80 *± 6,4* | 0,16 *± 0,1* | 0,72 *± 0,1* | 22,5 *± 7,7* | 0,12 *± 0,1* | 0,35 *± 0,2* |
| **Caecal tonsil** | | 21,05 *± 2,7* | 2,74 *± 1,3* | 47,48 *± 8,9* | 23,18 *± 2,8* | 2,33 *± 0,6* | 6,54 *± 1* | 23,45 *± 2,2* | 0,43 *± 0,1* | 0,86 *± 0,4* |
| **BM** | | 23,59 *± 2,3* | 1,15 *± 0,5* | 19,35 *± 5,8* | 23,23 *± 5,1* | 0,19 *± 0,03* | 0,51 *± 0,2* | 22,68 *± 4,7* | 0,09 *± 0,1* | 0,23 *± 0,2* |

## Supplementary Table 3

**Table S3.** Proportion of CD57^+^ cells among the Ig^+^ cells from different lymphoid organs based on the staining in Figure 6; (mean ± SD; n=5).

|  | **IgM** | **IgA** | **IgY** |
| --- | --- | --- | --- |
| **PBL** | 7,1 *± 3,3* | 33,4 *± 3,9* | 29,2 *± 19,9* |
| **spleen** | 4,1 *± 1,6* | 19,0 *± 7,9* | 21,4 *± 8,8* |
| **Caecal tonsil** | 5,2 *± 1,3* | 26,3 *± 5,9* | 34,9 *± 5,8* |
| **BM** | 5,8 *± 2,2* | 29,2 *± 10,4* | 25,0 *± 6,9* |

# Supplementary Figures

## Supplementary Figure 1

**
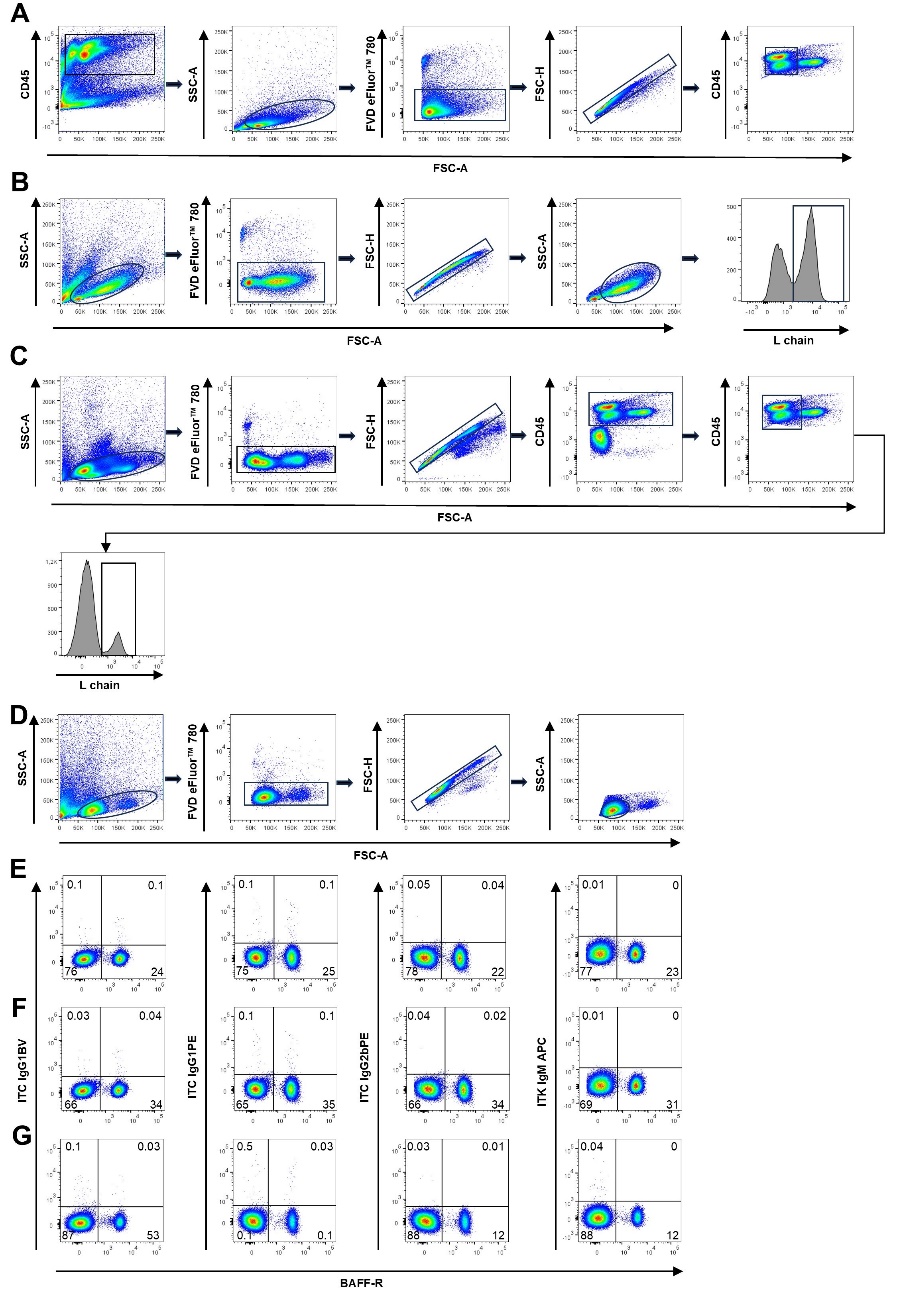
**

**Figure S1 Gating Strategies and Isotype controls**

In general, gating on CD45-positive cells was applied when analyzing different lymphoid organs (Figure 1 and 7). This approach was used because isolated cells from certain organs, particularly the bone marrow, typically contain a large proportion of non-leukocytes.

As splenocyte preparations usually consist almost exclusively from leukocytes, CD45 staining was omitted with the exception of IBDV vaccinated birds (Fig. 9) as the strong reaction to the applied live vaccines often leads to a higher content of nucleated erythrocytes in leukocyte preparations from spleen and blood.

As we could show that during differentiation B remain L chain positive, additional gating for L chain positive cells was applied to phenotyping experiments of splenocyte cultures.

(A) Gating strategy employed for Figure 1. Initially, a gate was established for CD45-positive cells, followed by subsequent gating for leukocytes in the scatter plot, exclusion of dead cells, and finally, gating for single cells. For PBMCs, an additional exclusion step for monocytes by size was included.

(B) Demonstrates the gating strategy utilized for splenic cell culture. Initially, the gate was set for leukocytes, followed by exclusion of dead cells and gating for single cells. Subsequently, single cells were further categorized into small and proliferating cells. Within the proliferatig cell population, an additional gate was established for light chain-positive cells. This gating strategy is depicted on cells stimulated with CD40L and IL-10 after 96 hours of stimulation. For the assessment of unstimulated splenic leukocytes, the fourth step of this strategy was omitted, as no proliferating cell population was observed.

(C) Depicts the gating used in the vaccination trial (Fig. 9). The first gate was set on leukocytes followed by dead cell exclusion, single cell gating, and exclusion of thrombocytes by their lower expression of CD45. Monocytes were excluded via cell size. Additional gating for light chain-positive cells was added when needed.

Isotype controls for sIg staining (IgG1, IgG2b) and CD57 (IgM) staining are displayed for bone marrow (D), spleen (E), and PBMCs (F). Blots are gated for viable single cells.

## Supplementary Figure 2

**
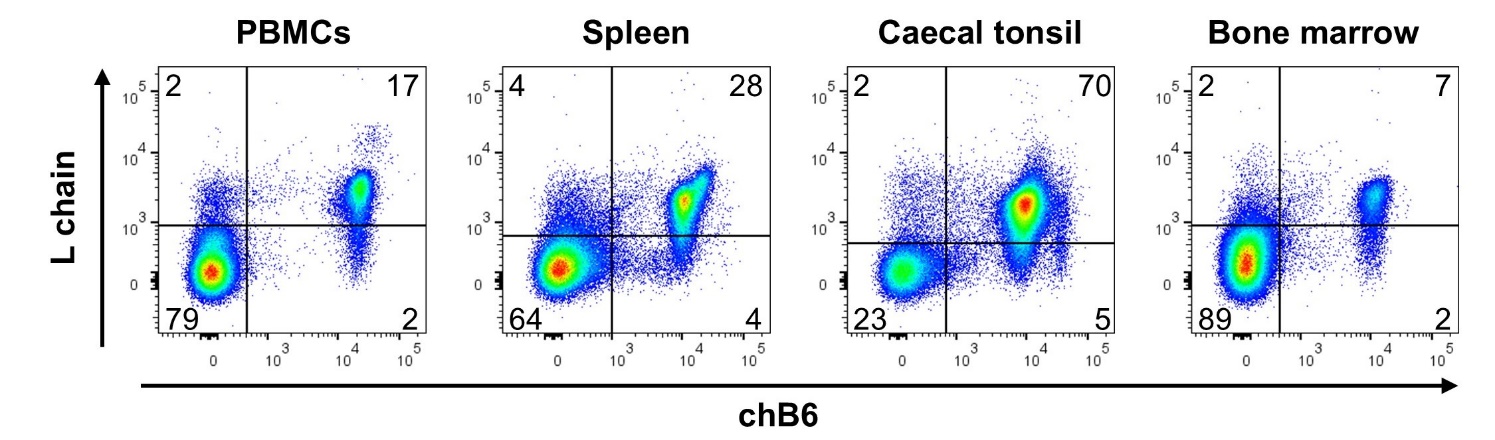
**

**Figure S2 L chain expression against chB6**

Leukocytes from the respective tissues of a young adult chicken (5,5 month) were stained with antibodies for L chain and chB6 and analyzed by flow cytometry.

Blots were gated for viable single lymphocytes. Data are representative for two independent experiments.

## Supplementary Figure 3


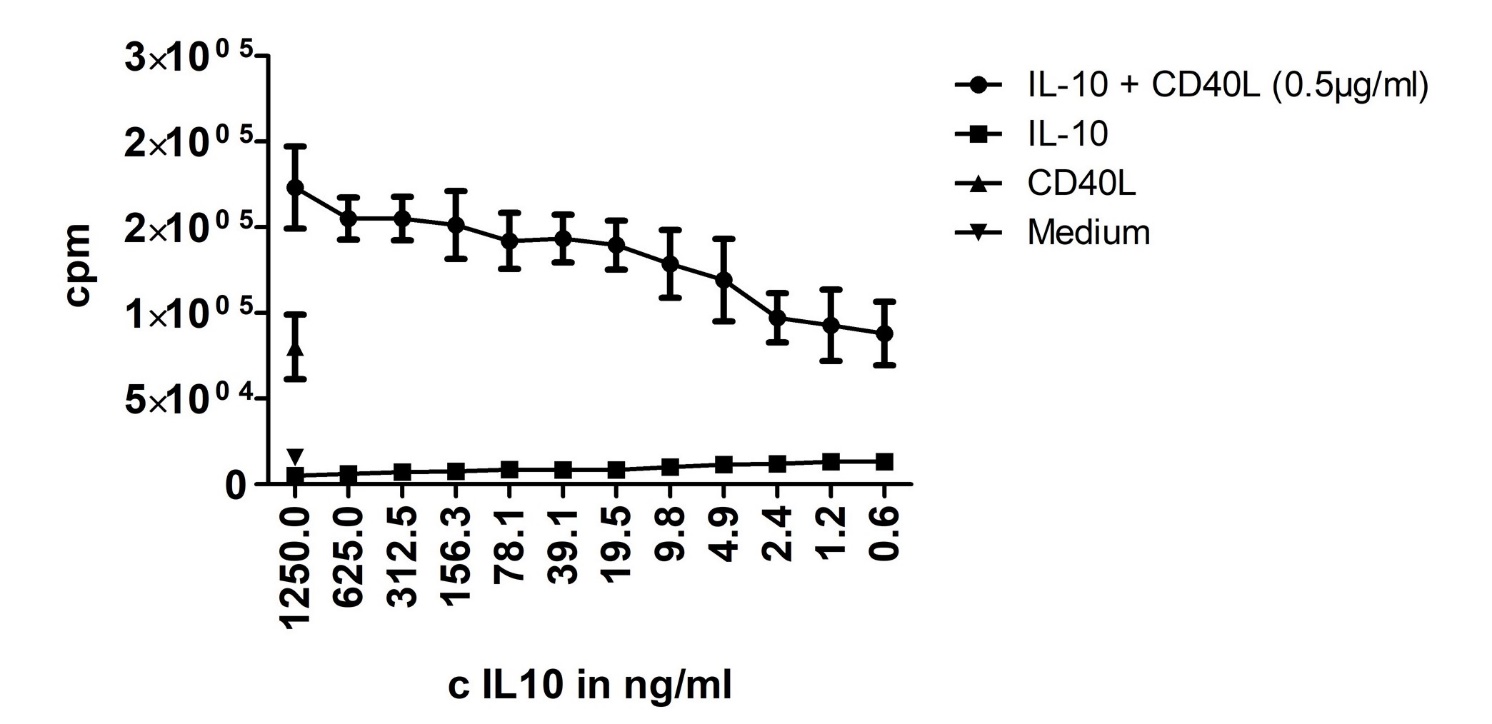


**Figure S3 Dose dependent effect of IL-10**

Leukocytes were isolated from spleen and incubated for 48h in the presence of CD40L, IL-10, a combination of both cytokines as well as medium only. In the combined stimulation, CD40L concentration was kept constant at 0,5µg/ml, while IL10 was serially diluted. After 16h cells were labeled with [³H]-Thymidine and harvested 24h later. Shown are mean ± SD of three experiments.

## Supplementary Figure 4


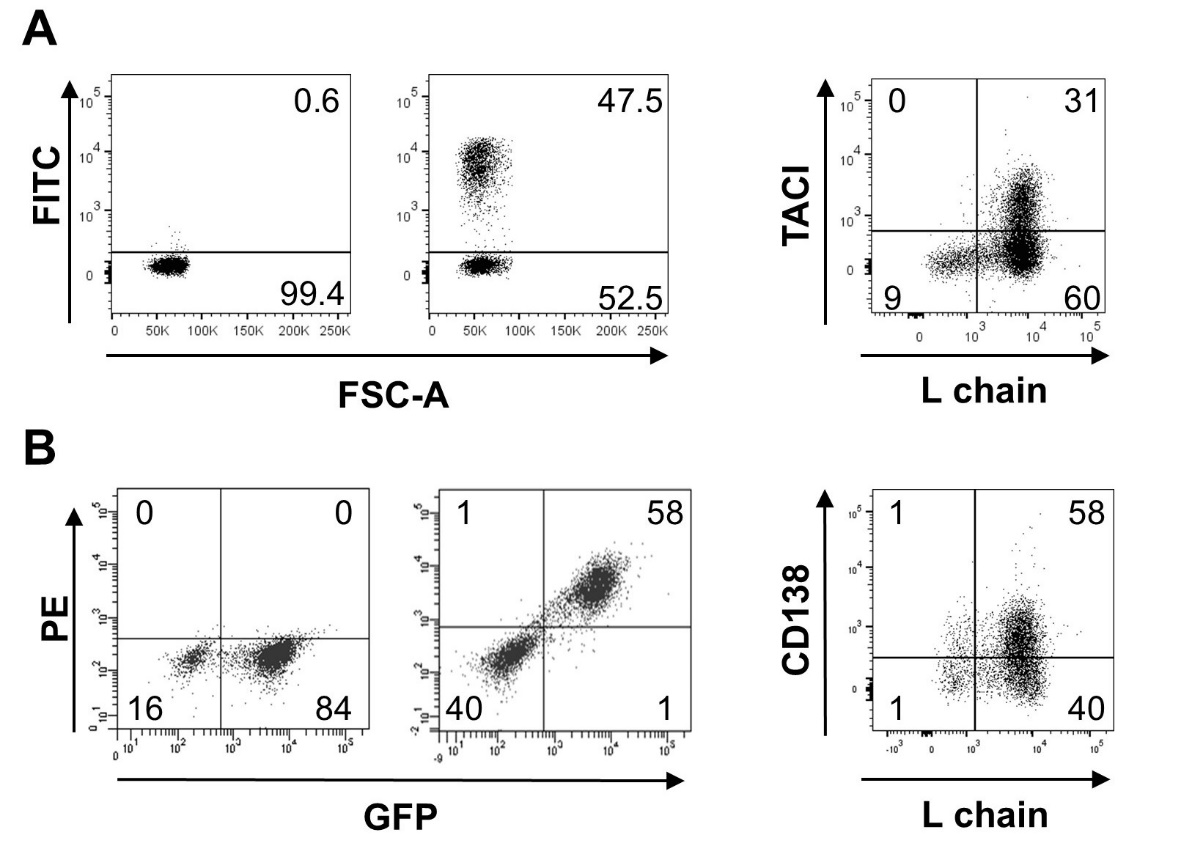


**Figure S4 Clone testing on transfected 293 cells**

(A) Illustrates the evaluation of the preselected TACI clone on stably transfected 293 cells; with the negative control depicted on the left and the preselected clone tested on a mixture of 293 and stably transfected 293-TACI cells. The dot blot on the right displays the staining of the selected clone on splenic leukocytes stimulated for 48 hours.

(B) Illustrates the testing of the preselected CD138 antibody clone on a mixture of 293-chCD138GFP cells with untransfected 293 cells, with the negative control shown on the left. The dot blot on the right displays the staining of the selected clone on splenic leukocytes stimulated for 48 hours.

Blots are gated for viable single cells.

## Supplementary Figure 5


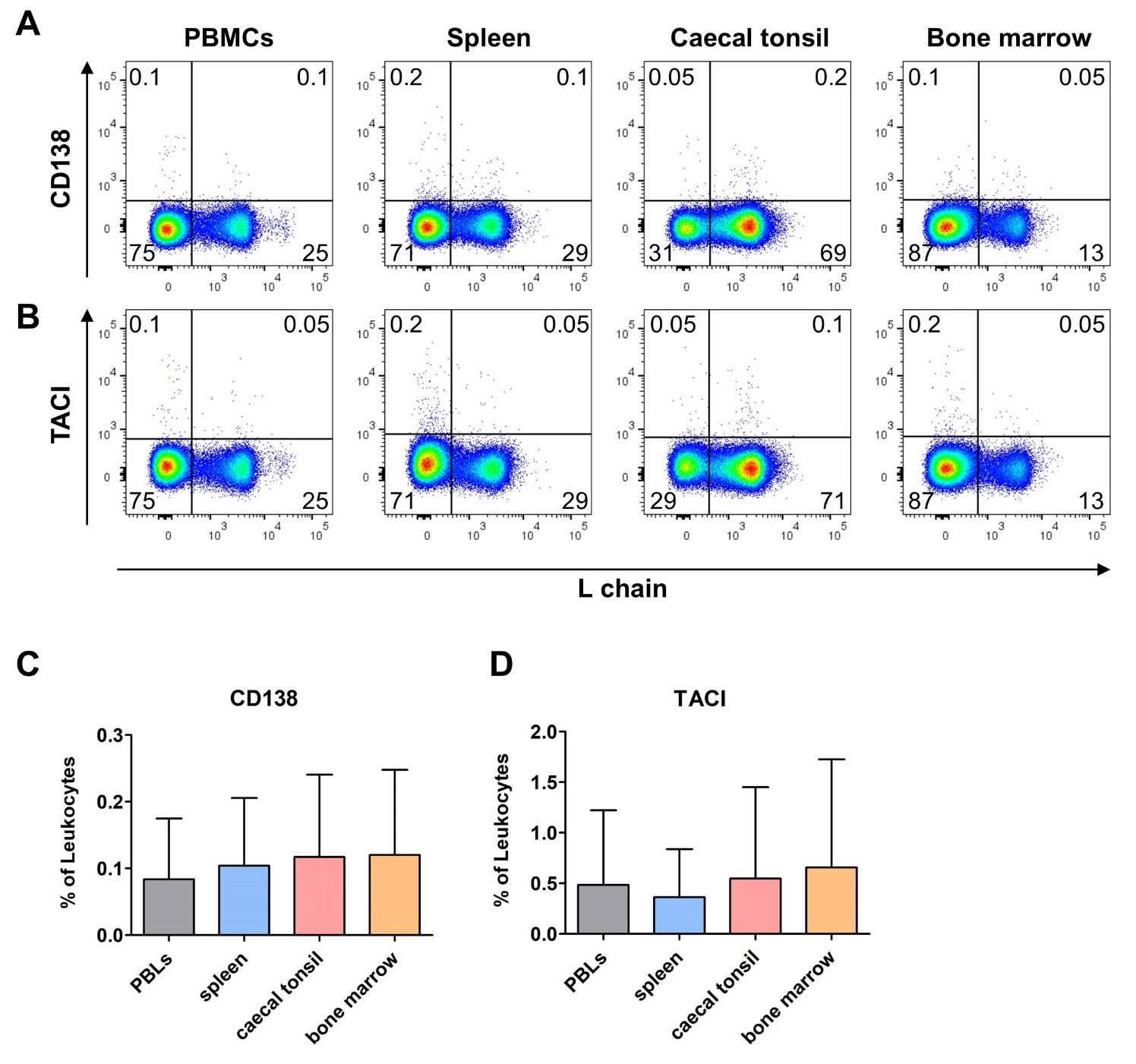


**Figure S5: TACI and CD138 expression in leukocytes**

Blots exemplify the expression of chCD138 (A) and chTACI (B) on CD45^+^ viable single cells isolated from different organs.

Bar graphs depict the frequency of chCD138 (C) and chTACI (D) on CD45^+^ viable single cells. Shown are mean ± SD of four experiments.

## Supplementary Figure 6


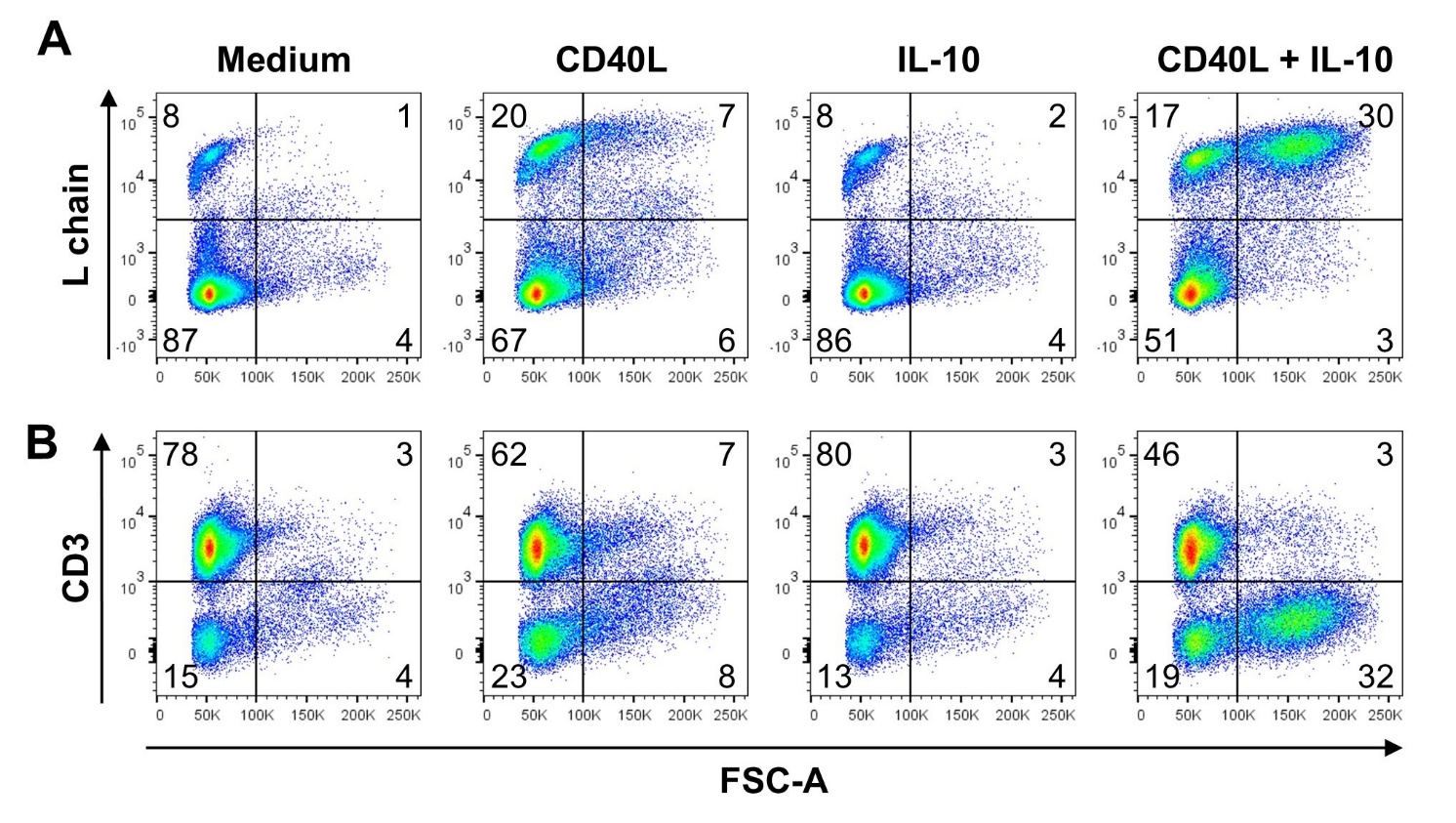


**Figure S6 Blast induction by different cytokines**

Depicts live single cells of 72h stimulated splenic leukocytes (shown in Figure 1A). Size of the cells is shown against their surface light-chain (A) and CD3 (B) expression. Blots are gated for viable single cells.

## Supplementary Figure 7


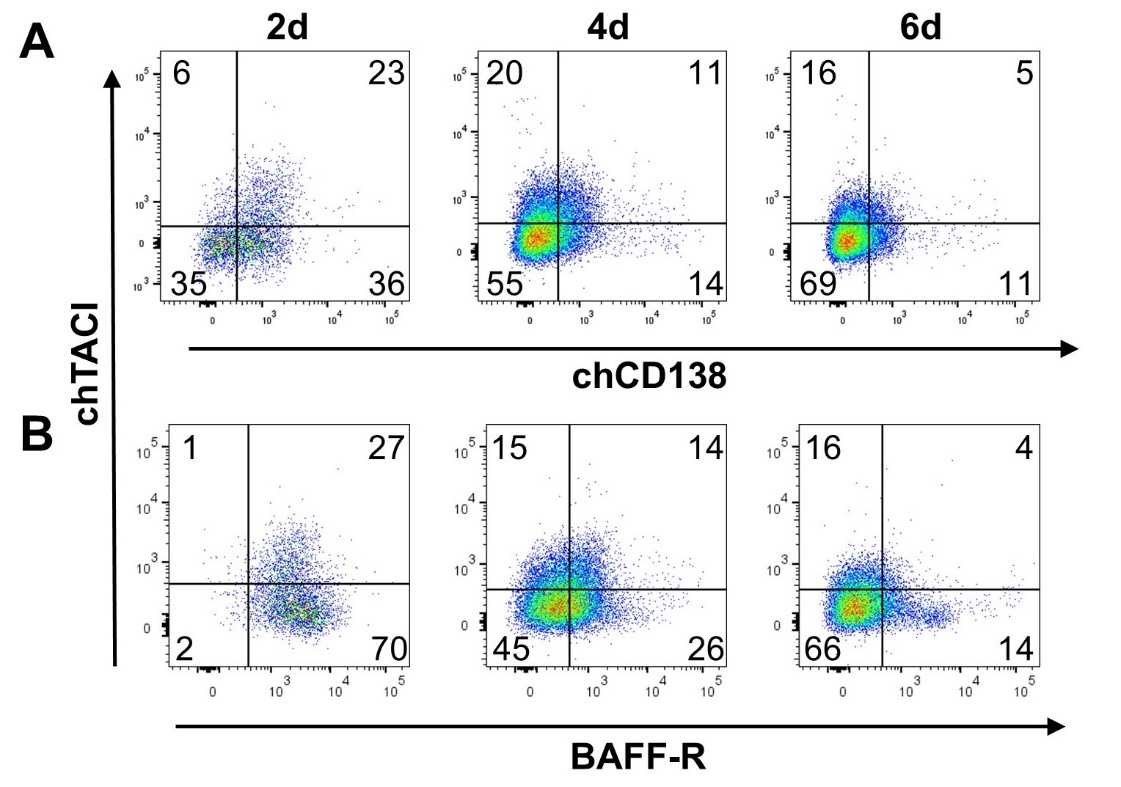


**Figure S7 Coexpression of TACI and CD138/BAFF-R**

Co-expression of TACI and CD138 (A) and of TACI and BAFF-R on splenic leukocytes, stimulated with CD40L and IL-10 is displayed.

Blots are gated for proliferating viable single cells.
